# A facile route for concurrent fabrication and surface selective functionalization of cellulose nanofibers by lactic acid mediated catalysis

**DOI:** 10.1038/s41598-023-41989-3

**Published:** 2023-09-07

**Authors:** Abdolrahim A. Rafi, Rana Alimohammadzadeh, Angelica Avella, Tanel Mõistlik, Martin Jűrisoo, Andreas Kaaver, Cheuk-Wai Tai, Giada Lo Re, Armando Cordova

**Affiliations:** 1 Department of Natural Sciences, Mid Sweden University, Holmgatan 10, 851 70 Sundsvall, Sweden; 2 Department of Industrial and Materials Science, Chalmers University of Technology, Rännvägen 2A, 41258 Göteborg, Sweden; 3 Department of Materials and Environmental Chemistry, Arrhenius Laboratory, Stockholm University, 10 691 Stockholm, Sweden

**Keywords:** Catalysis, Chemical engineering, Green chemistry, Materials chemistry

## Abstract

Celulose nanofibers are lightweight, recycable, biodegradable, and renewable. Hence, there is a great interest of using them instead of fossil-based components in new materials and biocomposites. In this study, we disclose an environmentally benign (green) one-step reaction approach to fabricate lactic acid ester functionalized cellulose nanofibrils from wood-derived pulp fibers in high yields. This was accomplished by converting wood-derived pulp fibers to nanofibrillated “cellulose lactate” under mild conditions using lactic acid as both the reaction media and catalyst. Thus, in parallel to the cellulose nanofibril production, concurrent lactic acid-catalyzed esterification of lactic acid to the cellulose nanofibers surface occured. The direct lactic acid esterification, which is a surface selective functionalization and reversible (de-attaching the ester groups by cleavage of the ester bonds), of the cellulose nanofibrils was confirmed by low numbers of degree of substitution, and FT-IR analyses. Thus, autocatalytic esterification and cellulose hydrolysis occurred without the need of metal based or a harsh mineral acid catalysts, which has disadvantages such as acid corrosiveness and high recovery cost of acid. Moreover, adding a mineral acid as a co-catalyst significantly decreased the yield of the nanocellulose. The lactic acid media is successfully recycled in multiple reaction cycles producing the corresponding nanocellulose fibers in high yields. The disclosed green cellulose nanofibril production route is industrial relevant and gives direct access to nanocellulose for use in variety of applications such as sustainable filaments, composites, packaging and strengthening of recycled fibers.

## Introduction

It is of utmost importance to expand the use and application of materials derived from renewable natural resources, which can decrease the dependence on fossil-based materials and plastics, for addressing global environmental challenges on land and in the oceans. In this context, cellulose is the most abundant biomaterial on earth, which is synthesized by photosynthesis at a rate of between 1011 and 1012 tons/year^1^. This renewable polysaccharide can be converted into nanocellulose, which can be isolated as cellulose nanofibers (CNFs) and/or cellulose nanocrystals (CNCs)^1–5^. This has sparked an intense research interest in both academia and industry^1–12^. CNFs (5–60 nm width, 0.1–2 μm length) have important properties such as biodegradability, biocompatibility, high mechanical strength, high toughness and low density^1–5^. Thus, CNFs have great potential for usage in several applications in various fields such as nanocomposites (as a reinforcement agent)^6^, packaging^7^, printed electronics^8^, medicine^9^, barriers^10^ and paper^11^. CNFs has been fabricated from renewable resources (e.g. agricultural residues, wood, grass) by means of different methodologies. Mechanical agitation is a common method for CNF fabrication^12–15^. However, CNF production using mechanical fibrillation (*e.g.* from wood derived pulp fibers) is extremely energy intensive and requires high energy consumption (generally > 30,000 kWht^−1^, 700–1400 MJ kg^–1^)^3,4,16–18^. Chemical treatment could decrease energy usage for CNF production. For example, the required energy for CNF fabrication was extensively reduced to < 7 MJ kg^−1^ as first shown by Isogai and co-workers by using sequential oxidation (2,2,6,6-tetramethylpiperidine-1-oxyl (TEMPO), sodium hypochlorite (NaClO)) and homogenization^16,19^. However, issues such as using an active radical initiator and a non-selective chlorine-based oxidant are drawbacks for this approach especially in large scale fabrication. CNF production by combined enzymatic hydrolysis/homogenization treatment is also requiring a low energy consumption^19,20^. Among the vide supra described CNF production methods, enzymatic hydrolysis/homogenization and mechanical agitation have the least environmental impact. However, enzymatic hydrolysis is usually time-consuming and other factors such as the prize and stability, which leads to biocide addition, can be disadvantageous for the users^12,19,20^.

Inspired by our research on direct organocatalytic esterification and ring-opening polymerizations of cellulose with aliphatic and functional acids (e.g. stearic acid, palmitic acid, oleic acid and 5-hexynoic acid) and cyclic esters (e.g. ε-caplolactone, lactide)^21–23^, we recently disclosed a selective and reversible esterification route for environmentally benign fabrication of CNFs in high yields using organic acids, which can be recycled, as reaction media and catalysts^18,23^. For example, formic acid was used for simultaneous hydrolysis and esterification to fabricate formic acid esterified nanocelluloses, which next could be deacylated by alkaline ester hydrolysis^18,24^. Later other studies revealed the successful fabrication of functionalized CNCs using oxalic acid or citric acid as the reaction media with or without a catalytic amount of HCl^25–27^. In addition, formic acid-esterified CNCs can be fabricated in neat formic acid using HCl as a co-catalyst^28^. Other organic acids such as citric acid^26,27^, benzoic acid^30^, 2-phenyl acetic acid^30^, lactic acid^31^ and acetic acid^32^ have also been used as the neat reaction media (no solvent) for fabrication of organic acid-grafted CNCs generally using a strong mineral acid as catalyst (Fischer esterification). Functionalization of celluloses are beneficial since it can improve the thermal stability and dispersion of the nanocellulose in polymeric matrixes^1^, open up for further functionalization as well as novel applications and click chemistry^3,18,32–35^.

In 1780, lactic acid (LA) was isolated from sour milk for the first time by Scheele and has a big research interest both in industry and academia^36^. Lactic acid is an organic acid that is biodegradable and relatively inexpensive and is produced on a large scale. Lactic acid is environmentally friendly since it would have less environmental impact during its disposal and it could be produced via microbial fermentation of renewable resources^37^. In addition to the above advantages, LA-esterification of nanocelluloses could make them more applicable and compatible with biodegradable polymers. As described vide supra, one approach to reach this nanocellulose products would be the concurrent LA esterification and nanocellulose fabrication using a strong mineral acid such as HCl as the catalyst. In this context, it was recently reported that lactic acid and oligo(lactic acid) functionalized CNCs can be produced from cotton linter using a mixture of lactic acid and concentrated HCl at 150 °C^31^. Moreover, post modification of CNFs with lactic acid ester groups was recently done using metal-based compounds as catalysts^38,39^. Strong acids such as HCl are hard to handle, corrosive and generate chlorine ions so these challenges become even more challenging and costly during scale-up. Based on our previous research, we became intrigued whether LA-modified nanocelluloses can be fabricated and esterified using a mild an organocatalytic and metal-free technology. Notably, it is believed that lactic acid itself is not strong enough to convert cellulose fibers into functional nanocelluloses. Moreover, we wanted to investigate if readily available wood-derived cellulose could be used as a substrate for the production of LA-modified nanocellulose. Herein, we disclose the LA autocatalyzed production and concurrent selective esterification of CNFs from wood-derived bleached sulphite pulp in high yields. The environmentally benign process does not need solvents or co-catalyst and the LA media was successfully recycled and reused for multiple production cycles providing the corresponding LA-esterified CNF in high yields (92%). In comparison, adding HCl as a co-catalyst decreased the yield of the nanocellulose process significantly (73%) and did not change the degree of substitution.

## Results and discussions

**Figure 1:**
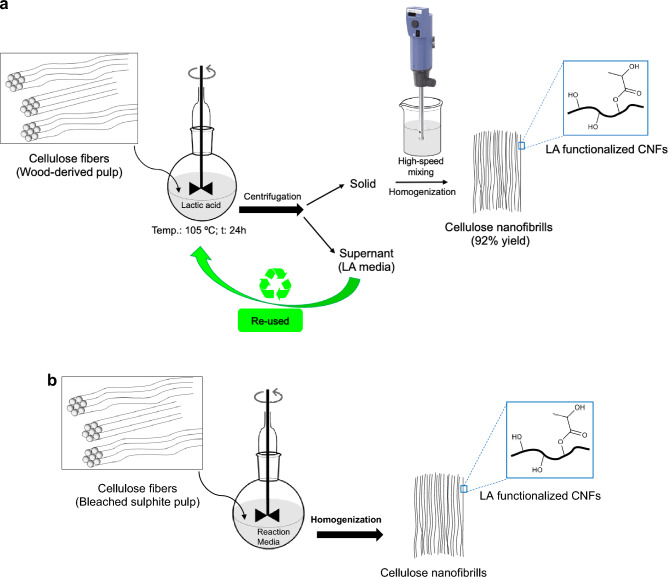
(**a**) Fabrication of lactic acid functionalized CNFs. (**b**) Fabrication of CNFs.

We began our investigation using bleached sulphite pulp as the substrate and lactic acid (*p*Ka of 3.86) as the neat reaction media (i.e. no solvent). In addition to discovering an organocatalytic nanocellulose fiber fabrication route with concurrent esterification, we wanted to develop a green process where the sustainable and biodegradable lactic acid could be successfully reused again (Fig. 1a).

The initial condition screening revealed to our delight that CNF can be produced in high yields using just lactic acid as the media (Table 1, Fig. 1b). For example, the yields of CNF when using just d,l-lactic acid (LA) or l-Lactic acid (LLA) were 92 and 91%, respectively (entries 1 and 2). We also observed that adding a co-catalytic amount of HCl significantly decreased the yields. In these cases, the yields using 0.05 and 0.1M HCl as the co-catalyst were 73 and 65%, respectively (entries 3 and 4). Thus, the disclosed sustainable one-step selective esterification and nanocellulose production method was advantageous, straightforward, and eco-friendly since it doesn’t need to use toxic and harsh reagents or conditions as well reduced manipulation steps. The CNFs degree of substitution (D.S.) of lactic acid was determined according to literature using alkaline titration^40^. In all the entries shown in Table 1, the D.S. was between 0.20 and 0.22. This degree of substitution corresponds to a degree of esterification on every fifth glucose unit and a selective surface functionalization. In addition, the esterification is reversible and the ester groups can be removed using alkaline conditions. This could be desirable for certain types of applications such as assembly of native elementary CNFs^35^. The results presented in Table 1 also demonstrate that LA-mediated concurrent hydrolysis and esterification, which is autocatalytic, of sulphite pulp is enough for generating CNFs.

**Table 1:** Fabrication of CNFs.

Entry	Reaction media^a^	Acid conc.^b^	Yields%^c^	d.s.^d^
1	LA	–	92	0.21
2	LLA	–	91	0.21
3	LA + HCl	0.05 M	73	0.20
4	LLA + HCl	0.1 M	65	0.22

(a) Temperature: 105 °C, Reaction time: 24 h. (b) Total Concentration of HCl in the reaction medium. (c) Isolated yields of pure CNF. (d) Degree of substitution determined according to the “Experimental section”.

Recycling of the reaction media and avoidance of waste generation are important factors in developing green chemistry and industrialized scale-up^39^. Thus, we investigatedthe recyclability of lactic acid-mediated nanocellulose production process (Table 2).

**Table 2:** Cycling and reusing of the LA media in the CNF fabrication.^a^

Cycles	Yields (%)	Yields (%)b
1st	92	73
2nd	91	77
3rd	91	82
4th	92	85
5th	91	83
6th	92	84
7th	92	85
8th	92	n.d.

^a^Bleached sulphite pulp (5 g) was inserted into a round-bottom flask and lactic acid (90%, 200 mL) was added. After stirring the reaction mixture with a mechanical stirrer (1400 rpm) at 105 °C for 24 h, it was cooled down to room temperature. The reaction mixture was next transferred to a tube and centrifuged (12,000 rpm) for 14 min. The CNFs were isolated and the supernatant was collected and reused as the reaction media for the next reaction cycle.

^b^HCl (37 wt.%, 4.2 mL, total concentration of 0.05 M) was also added. N.d., not determined.

We found that the catalytic lactic acid media could be recycled for multiple process cycles giving the corresponding lactic acid esterified CNF in high yields. In comparison, when adding a co-catalytic amount of HCl (0.05 M) the CNF is produced in lower yields. Moreover, the yield of the lactic acid modified CNF in the presence of HCl increased as the reaction cycles increases. This is due to decrease in acidity of the reaction media as can be noticed after the third cycle when starting with a co-catalytic amount of HCl. In addition, the recycling is readily performed at a 25-g scale maintaining a high yield of the generated nanocellulose (See SI, Table S1).

### FT-IR spectra

**Figure 2:**
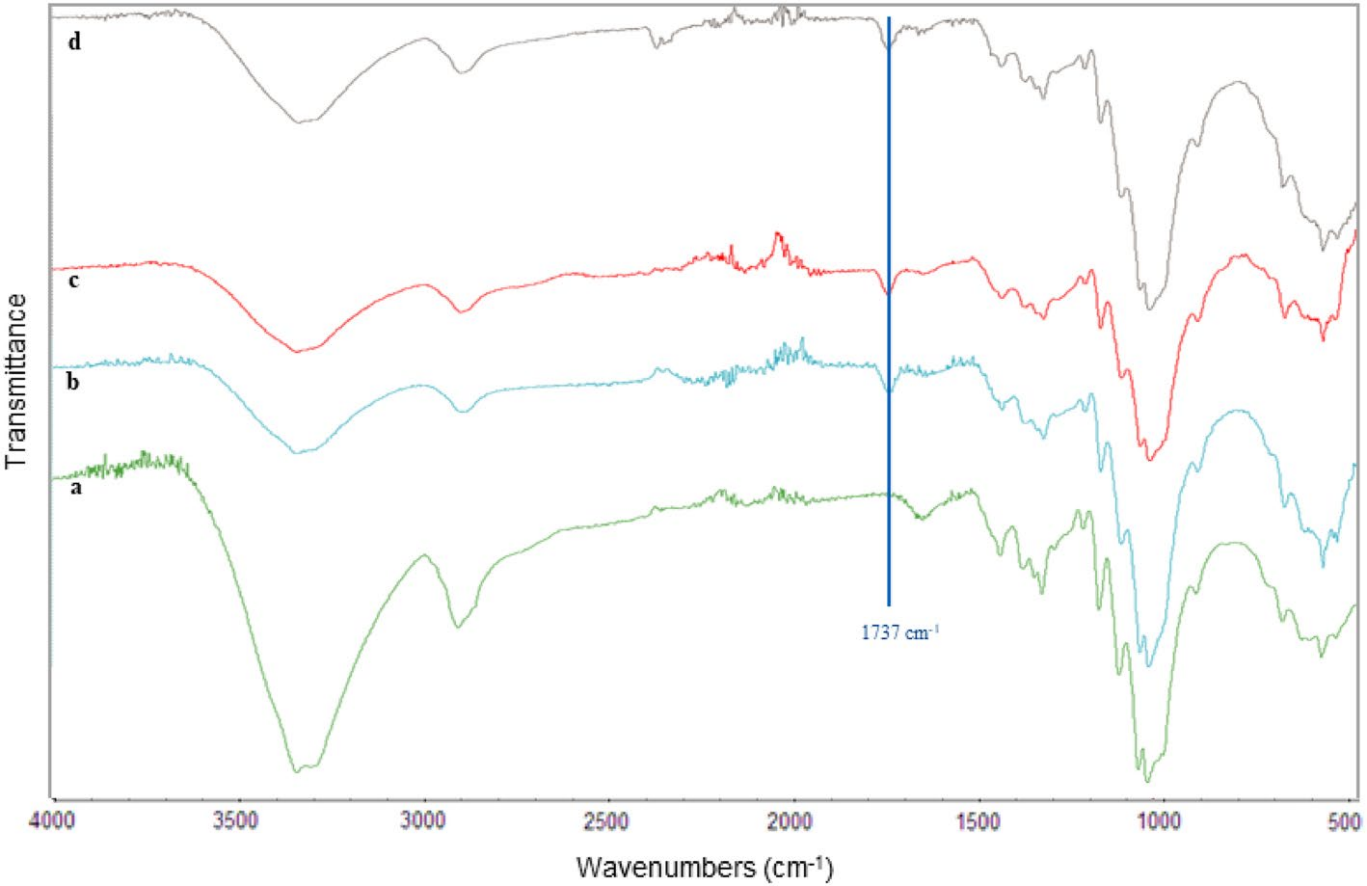
FT-IR spectra of (**a**) cellulose starting material sulphite pulp (spectrum a), (**b**) LA functionalized CNFs (in the presence of HCl 0.1 M), (**c**) LA functionalized CNFs (in the presence of HCl 0.05 M), and (**d**) LA functionalized CNFs (No added HCl).

The FT-IR spectra of bleach sulphite pulp (cellulose) and LA functionalized CNFs are presented in Fig. 2. The wide absorption peaks in all spectra around 3335 cm^−1^ and 2892 cm^−1^ are attributed to the O–H and C–H stretching vibrations, respectively. The absorption bands around 1638 cm^−1^ corresponds to the O–H bending vibrations of the hydroxyl groups of absorbed water. The absorption bands around 1161 cm^−1^ and 1103 cm^−1^ are attributed to the stretching vibrations of C–C and C–O, respectively. The absorption peaks around 1027 cm^−1^ comes from the vibration of C–O–C in the pyranose^42,43^. It is noteworthy that a new peak appears around 1737 cm^−1^ in the CNFs spectra, which corresponds to an ester bond. Thus, the presence of this peak confirms that lactic acid esterification of the hydroxyl groups of the CNFs had occurred. The same peak was also observed in the FT-IR spectra of the CNFs produced by using recycled LA media (see supporting information). This shows that successful direct selective esterification also occurred in these cases.

### Solid state CP/MAS ^13^C NMR

**Figure 3:**
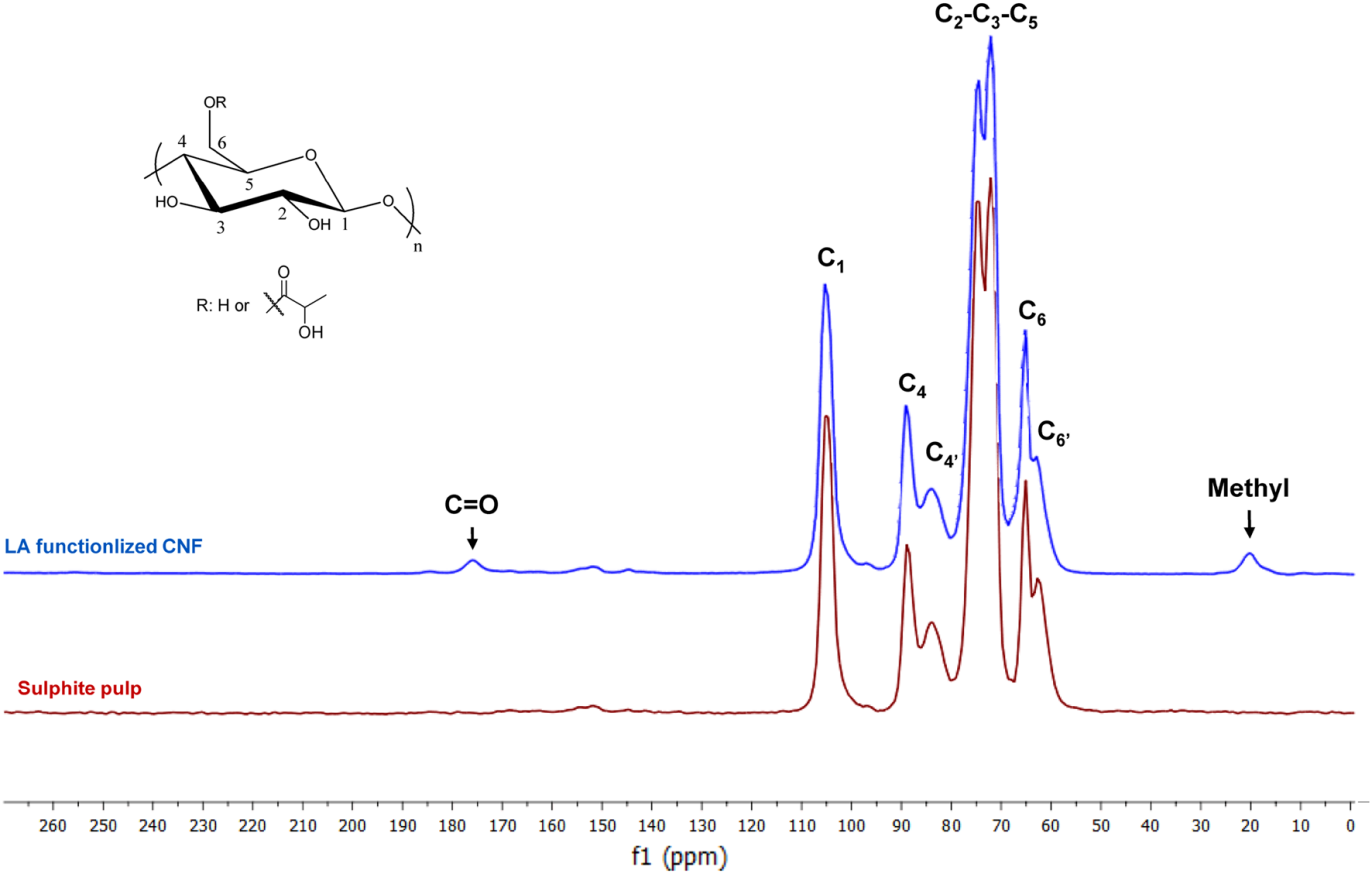
Solid state CP/MAS ^13^C NMR spectra; red spectrum for sulphite pulp as starting material, and blue spectrum for LA functionalized CNF (using lactic acid in the presence of HCl 0.1 M).

Figure 3 shows the solid-state CP/MAS ^13^C NMR spectra of sulphite pulp (starting material) and LA functionalized CNFs (using lactic acid and HCl 0.1 M). The sulphite pulp spectrum depicts the characteristic carbon resonances of cellulose at 105 ppm, 89 ppm, and 66 ppm for C1, C4, and C6, respectively. C4′ and C6′ are assigned to amorphous parts that come at 84 ppm and 63 ppm, whereas the C4 and C6 corresponds to the ordered cellulose structure. The cluster at 72–75 ppm belong to C2-C3-C5 in cellulose^44^. In comparison with sulphite pulp spectrum, LA functionalized CNF has two new signals at 20 ppm that belongs to –CH_3_ of LA, and 176 ppm is assigned to carbonyl group (CO) confirming the happening of the esterification reaction between LA and hydroxyl groups in CNFs. We did not observe any ester peaks corresponding to oligomeric lactide or poly(lactide. Moreover, the degree of substitution of the lactic acid esterified CNF is low (see Table 1) and according to the literature by Iversen the organic acid-catalyzed esterification is regioselective^45,46^. It is also in accordance with the proposal by Spinella et al.^29,31^.

Moreover, the crystallinity index was calculated from solid state CP/MAS ^13^C NMR spectra by dividing the area under C4 crystalline peak (C4 from 86.6 to 93 ppm) by the total area of C4 resonances from residual amorphous and crystalline domains (C4 + C4′ from 80 to 93 ppm)^47,48^. The crystallinity indexes of sulphite pulp and LA functionalized CNF (using LA and HCl 0.1 M) were in the same range and calculated to be 0.52 and 0.53, respectively. Since the crystallinity index is nearly the same, the process did not afford cellulose nanocrystals.

The average crystallite sizes of the sulphite pulp and the LA functionalized CNF (using LA and HCl 0,1 M) were calculated from their XRD spectra using the *Scherrer* equation (Eq. 1, section “X-ray diffraction (XRD) measurements”), and calculated to be 3.6 nm and 3.1 nm, respectively. Thus, they are in the same range as reported in the literature for *Norway spruce Picea abies* (L.) *Karst*. (3.2 nm), and *Scots pine Pinus sylvestris* L. (3.1 nm), respectively^49^.

### Morphology and microscopy studies

**Figure 4:**
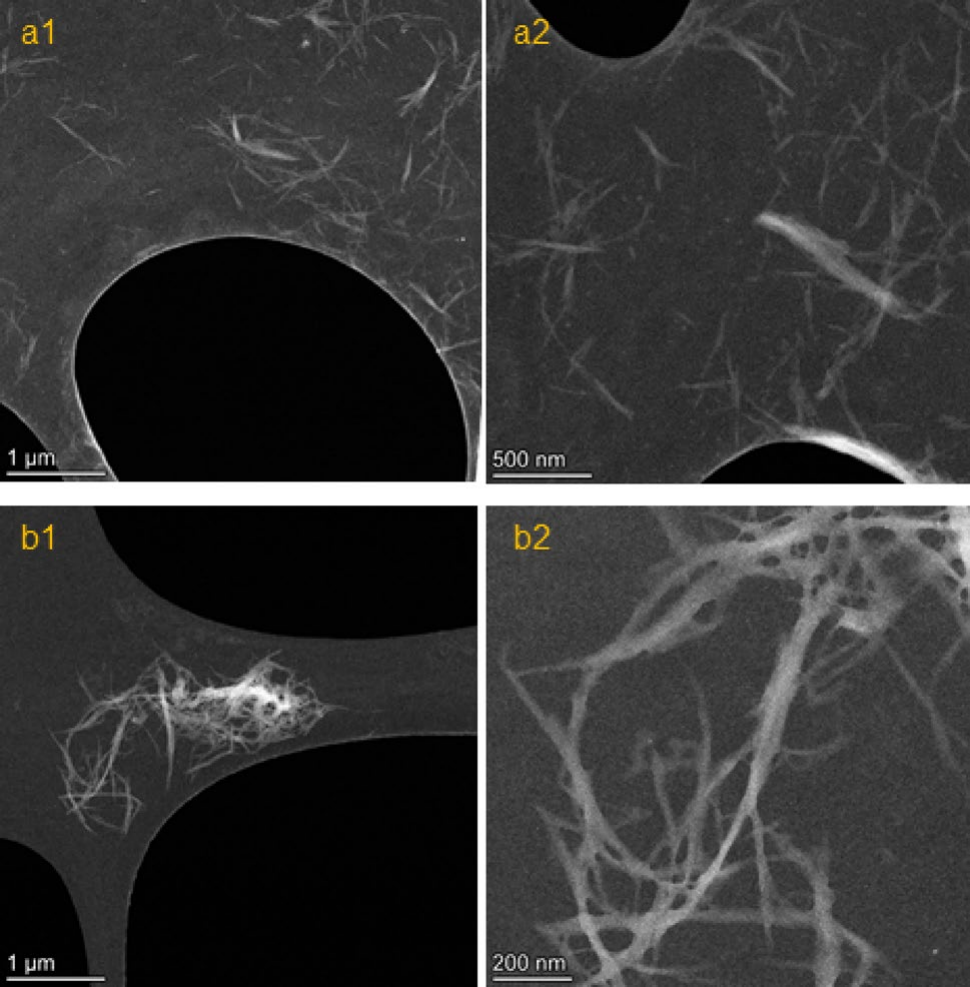
HAADF-STEM images of LA functionalized CNFs; (**a1, a2**) fabricated with lactic acid and HCl 0.1 M, and (**b1, b2**) fabricated with lactic acid (without adding HCl).

Figure 4, depicts the HAADF-STEM images of LA functionalized CNFs, fabricated using lactic acid and HCl 0.1 M (a1, a2) or just lactic acid (b1, b2), respectively, in different magnifications. According to the STEM images, the fabricated LA functionalized CNFs have nano-size dimensions. Moreover, the AFM image of LA functionalized CNFs fabricated with lactic acid (without adding HCl), and the SEM images (of starting material, and LA functionalized CNFs) were shown in Figs. S29 and S32, respectively. The aspect ratio for LA functionalized CNFs fabricated with lactic acid (without adding HCl) was calculated to be 162 ± 24 by measuring of around 100 different individualized CNF with an average length of 1236 ± 214 nm and diameter of 8 ± 2 nm).

### Thermal analysis

**Figure 5:**
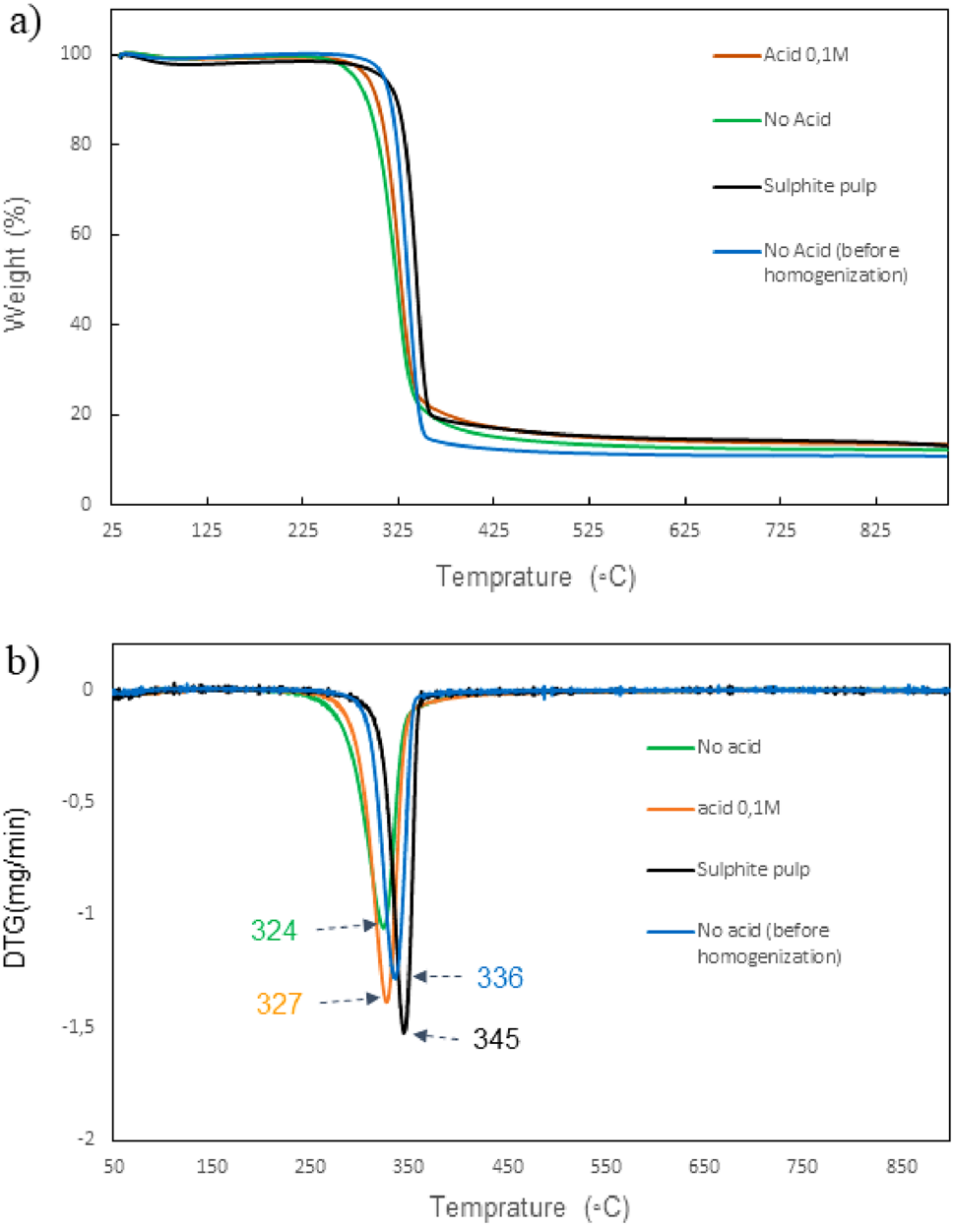
(**a**) TGA curves of sulphite pulp, LA functionalized CNFs fabricated in the presence of HCl 0.1 M, with no added HCl, and no added HCL before homogenization. (**b**) DTG curves of the related samples.

TGA and DTG curves of the cellulose sulphite pulp, LA-functionalized CNF fabricated by LA and HCl (0.1 M) co-catalysis and LA-functionalized CNF fabricated by autocatalysis (no acid) as well as the latter material before the homogenization step are depicted in Fig. 5. Two main weight losses for each sample were observed for the TGA curves. The initial weight loss (around 80 °C) was attributed to the evaporation of water from the samples. Next, a significant weight loss owing to the decomposition and degradation of the sample was observed. No big differences between the TGA curves related to the LA-functionalized CNF was observed; however, the thermal stability of the CNF is slightly lower than that of the starting sulphite pulp. Moreover, the thermal analysis of the samples fabricated by autocatalysis procedure (no addition of HCl) and taken before and after homogenization are slightly different. In fact, the degradation temperature of the samples after homogenization is slightly lower (324 °C) than that of the sample before the homogenization (336 °C). The lower degradation temperature of the prepared CNF samples as compared to the starting material could be due to the smaller fiber dimensions of the CNF as compared to that of sulphite pulp^50^.

### Molecular weight distribution

The molecular weight distribution and degree of polymerisation (DP) of the LA functionalized CNF fabricated by autocatalysis was determined by size exclusion chromatography (SEC). The average M_w_, M_n_, PDI (poly dispersity index) and DP (degree of polymerization) were 112,639 g mol^−1^, 21,142 g mol^−1^, 5.3 and 131, respectively. This is very similar to the average molecular weight (M_w_) of the formic acid generated CNF, which has a M_w_ of 112,360 g mol^−1^ a PDI of 6.8^18^.

In addition, the average Mw of the sulphite pulp (made of 70% fresh *Norway spruce (Picea abies*) and 30% *Scot pine (Pinus sylvestris)*) is 549,987 g mol^−1^ with a PDI of 10.7.

### The effect of lactic acid modification

We also investigated the effect of lactic acid modification. Thus, we prepared films from the LA functionalized CNF and just CNF obtained after removal of the lactic acid groups by alkaline hydrolysis, respectively. Next, we investigated the mechanical properties of the film. We found that the strength properties slightly increased by the lactic acid modification (Table 3). We also investigated the contact angle for the LA-CNF-derived and CNF-derived film, respectively (See supporting information). The contact angles were 58 ± 1° for the LA-CNF film and 42 ± 3° for the CNF film. Thus, lactic acid modification of the CNF slightly improved the contact angle.

**Table 3:** Mechanical data of the prepared films at 50% RH.

Sample	E-modulus [GPa]	Strain at break [%]	Tensile strength [MPa]
LA-CNF	5.7 ± 0.3	1.3 ± 0.3	42 ± 1
CNF (hydrolysed LA-CNF)	5.1 ± 0.4	1.3 ± 0.2	39 ± 2

We also investigated the effect of adding CNF and LA modified CNF to poly(lactic acid). We found that both increased the strength of the PLA (See supporting information). With CNF, giving the highest improvement of the strength.

## Conclusions

Environmentally benign methods for producing nanocellulose fibers in large scale is very important. Moreover, selective functionalization of cellulose with lactic acid ester groups gives “cellulose lactate”, which can be converted to nanocellulose fibers with improved compatibility with other biodegradable polymers (e.g. poly(lactic acid), poly(ε-caprolactone)) and plastics. Herein, we present a scalable and selective route for the concurrent direct esterification and fabrication of CNFs from wood-derived pulp in high yields using lactic acid as reaction media and catalyst. The disclosed lactic acid-media for the nanocellulose fabrication process is recyclable and can be used for multiple cycles at large scale without effecting the yield or degree of substitution of the produced nanocellulose lactate fibers. Thus, the disclosed method full-fills important green chemistry criteria (minimizing waste, high selectivity, no toxic chemicals, sustainable starting material and product, recyclable, atom economic, low energy, high yielding, one-step)^51^, is industrially relevant^41^ and is an eco-friendly process for fabricating biodegradable functionalized CNF for potential industrial usage in variety of applications (e.g. sustainable filaments, composites, packaging, nonwoven materials, and strengthening of recycled fibers).

## Experimental section

### Materials

Bleached sulphite dissolved softwood pulp (70% Norway spruce (*Picea abeis*) and 30% Scots pine (*Pinus sylvestris*)) was received from Domsjö Fabriker AB (Sweden). d,l-Lactic acid (90%), l-Lactic acid (98%) and Hydrochloric acid (37 wt%) were purchased from VWR BDH chemicals. All chemicals were used as received without further purification.

### Solid state CP/MAS ^13^C NMR

Solid state NMR spectra were recorded by means of a Bruker Avance III 500 MHz spectrometer equipped with a 4 mm HX CP MAS probe. Experiments were acquired at a magic angle spinning (MAS) rate of 10 kHz and the temperature 298 K. The cross-polarization (CP) experiments used a 90° excitation pulse of 3 us for 1H, followed by a contact time of 1.5 ms with a ^13^C spin lock frequency of 60 kHz while 1H was ramped from 45 up to 90 kHz. The 1H decoupling scheme at 83 kHz was applied during the acquisition. The relaxation delay was 2 s and Adamantane was used as an external reference with the CH_2_ signal at 38.48 ppm.

The crystallinity index was determined by separating the C4 region of the spectrum into amorphous and crystalline peaks, then dividing the area under the C4 crystalline peak (86.6–93 ppm) by the total area of C4 resonances from residual amorphous and crystalline domains (80–93 ppm)^47,48^.

### Fourier transform infrared spectroscopy (FT-IR)

Thermo Scientific NICOLET 6700 FT-IR (Smart orbit, Diamond 30,000–200 cm^−1^) was used to record the FT-IR spectra*.*

### Transmission electron microscopy (TEM)

All TEM experiments were carried out on a 200 kV JEOL JEM-2100F field-emission electron microscope equipped with an ultra-high resolution pole piece (Cs = 0.5 mm). High-angle annular dark-field images were acquired by JEOL ADF detector using Gatan DigitalMicrograph. The incident beam probe was 0.2 nm with convergence angle of ~ 10 mrad and the camera length of 8 cm were used. The samples were dispersed on Cu TEM grid with holey carbon supporting films.

### X-ray diffraction (XRD) measurements

X-ray diffraction (XRD) measurements were performed using “Bruker AXS D2 Phaser”. The X-ray generator was equipped with a Cu tube operating at 30 kV and 10 mA and irradiating the sample with a monochromatic CuKα radiation with a wavelength of 1.54 Å. XRD spectra were acquired at room temperature over the 2θ range of 5°–50° with sampling at 0.03° increments and with a measurement time of 1 s per 2θ intervals. The average crystallite size was calculated from the *Scherrer* equation (Eq. 1), by using the diffraction pattern obtained from the 002 lattice planes of cellulose.

1 $${\text{D }} = \, \left( {{57}.{\text{3k}}\lambda } \right)/\left( {\beta {\text{cos}}\theta } \right)$$

where: D is the mean diameter of crystal, k is the crystal shape factor (0.94), λ is the X-ray wavelength (1.54 Å), β is the FWHM (full width at half maximum) of peak diffraction corresponding to the crystallographic plane 002, and θ is the Bragg angle corresponding to the (002) plane. A factor of 57.3 was applied in the equation in order to convert β from degrees to radians.

### Typical lactic acid mediated synthesis of LA functionalized CNFs.

To a round-bottom flask (500 mL), bleached sulphite pulp (5 g) and lactic acid (90%, 200 mL) were subsequently added. After stirring the reaction mixture with a mechanical stirrer (1400 rpm) at 105 °C for 24 h, the reaction temperature was decrease to room temperature and the reaction mixture transferred to a centrifuge vial (250 mL). Centrifugation (12,000 rpm, 14 min) was followed by separation of the supernatant, which was reused for additional reaction runs (recycling), and the solid material was collected. The collected solids were re-dispersed in distilled water and washed by centrifugation (3 × 200 mL H_2_O). Next, the washed solid cellulose was dispersed into distilled water (200 mL) and homogenized (IKA® T25 ULTRA TURAX, 14,000 rpm) for 90 min. To determine the yield of the CNF, the suspension was centrifuged (12,000 rpm, 14 min) and next the water was decanted. The CNF solids were collected and lyophilized. The lyophilized solid CNF was broken down into a powder using mortar and pestle and dried 18 h under reduced pressure. The yield of the CNF was 92% (4.6 g). The average Mw was determined by SEC (Mw = 112,639 g mol^−1^, PDI = 5.3).

### Typical lactic acid mediated synthesis of LA functionalized CNFs (HCl, 0.05 M)

To a round-bottom flask (500 mL), bleached sulphite pulp (5 g), lactic acid (90%, 200 mL) and HCl (0.84 mL, 37 wt.%, total concentration 0.05 M) were subsequently added. After stirring the reaction mixture with a mechanical stirrer (1400 rpm) at 105 °C for 24 h, the reaction temperature was decrease to room temperature and the reaction mixture transferred to a centrifuge vial (250 mL). Centrifugation (12,000 rpm, 14 min) was followed by separation of the supernatant, which was reused for additional reaction runs (recycling), and the solid material was collected. The collected solids were re-dispersed in distilled water and washed by centrifugation (3 × 200 mL H_2_O). Next, the washed solid cellulose was dispersed into distilled water (200 mL) and homogenized (IKA® T25 ULTRA TURAX, 14,000 rpm) for 90 min. To determine the yield of the CNF, the suspension was centrifuged (12,000 rpm, 14 min), water was decanted and the solids were lyophilized. Next, the lyophilized solid CNF was broken down into a powder using mortar and pestle and dried under reduced pressure for 18 h. The CNF yield was determined 73% (3.65 g).

### Synthesis of LA functionalized CNFs (in the presence of HCl, 0.1 M)

To a round-bottom flask (500 mL), bleached sulphite pulp (5 g), lactic acid (90%, 200 mL) and HCl (1.7 mL, 37 wt.%, total concentration 0.1 M) were subsequently added. After stirring the reaction mixture with a mechanical stirrer (1400 rpm) at 105 °C for 24 h, the reaction temperature was decrease to room temperature and the reaction mixture transferred to a centrifuge vial (250 mL). Centrifugation (12,000 rpm, 14 min) was followed by separation of the supernatant, which was reused for additional reaction runs (recycling), and the solid material was collected. The collected solids were re-dispersed in distilled water and washed by centrifugation (3 × 200 mL H_2_O). Next, the washed solid cellulose was dispersed into distilled water (200 mL) and homogenized (IKA® T25 ULTRA TURAX, 14,000 rpm) for 90 min. To determine the yield of the CNF, the suspension was centrifugated (12,000 rpm, 14 min) and the water was decanted and the solids were collected by lyophilization. The lyophilized solid CNF was broken down into a powder using mortar and pestle and dried for 18 h under reduced pressure. The CNF yield was determined to 65% (3.25 g).

### Determination of Degree of Substitution (D.S.)

The titration method was used in order to determine the D.S. of samples. A 200 mg of sample was dispersed in 16 mL of EtOH (*aq*) 70% v/v, then 8 mL of NaOH (0.5 mol L^−1^) was added and the mixture was stirred at 60 °C for 24 h. After cooling down to room temperature, 4 − 3 drops of phenolphthalein indicator was added, and the solution was titrated against 0.5 mol L^−1^ HCl solution. The DS values were calculated via the Eq. (2):

2 $${\text{D}}.{\text{S}}. \, = {\text{ 162 M }}\left( {{\text{V}}_{0} - {\text{V}}} \right) \, /{ 1}000{\text{ W}}$$

where: 162 is the molecular mass of an AGU (anhydro glucose unit), V_0_ is the volume of HCl solution (mL) consumed for titration of the blank sample (reference), V is the volume of HCl solution (mL) used for titration of the sample, M is the molarity of HCl solution, and W is the weight of sample (g).

### Thermogravimetric analysis (TGA)

Thermogravimetric analysis (TGA) was carried out under nitrogen gas with flow rate of 75 mL min^−1^, with a scan rate of 5 °C min^−1^, using a Mettler Toledo TGA/DSC 1.

### Molecular weight distribution

The sample was dissolved in 8% (w/v) LiCl in DMAc and before analysis the sample was diluted to 1.6%. PL-GPC 220 with RI-detector as well as the following conditions were used for the analysis. Columns: 20 µm Mixed-A columns from polymer Lab., one guard column and two 30 cm columns in series; Standards: the pullulan standards from polymer Lab with molar weights of 708 KDa, 344 KDa, 200 KDa, 107 KDa, 47.1 KDa, 21.1 KDa, 9.6 KDa, and 6.1 KDa were used; The flow rate was 1 mL min^−1^. Temperature: 70 °C; and the eluent was 0.5% (w/v) LiCl in DMAc.

### Supplementary Information

Supplementary Information.

## Data Availability

All data generated or analyzed during this study are included in this published article [and its supplementary information files].
