# Supplementary material for: A facile route for concurrent fabrication and surface selective functionalization of cellulose nanofibers by lactic acid mediated catalysis
Source: Sci Rep. 2023 Sep 7;13:14730. doi: 10.1038/s41598-023-41989-3 (PMC10484996; doi:10.1038/s41598-023-41989-3)
Supplement: Supplementary file 1 — Supplementary Information. [file 41598_2023_41989_MOESM1_ESM.pdf]

Supporting Information:

A facile route for concurrent fabrication and surface selective functionalization of cellulose nanofibrils by lactic acid mediated catalysis

Abdolrahim A. Rafi[1], Rana Alimohammadzadeh[1], Angelica Avella[2], Tanel Mõistlik[1], Martin Jűrisoo[1], Andreas Kaaver[1], Cheuk-Wai Tai[3], Giada Lo Re[2] and Armando Cordova[1,*]

[1)] Department of Natural Sciences, Mid Sweden University, Holmgatan 10, 851 70 Sundsvall, Sweden.

[2)] Department of Industrial and Materials Science, Chalmers University of Technology, Rännvägen 2A, 41258 Göteborg, Sweden.

[3)] Department of Materials and Environmental Chemistry, Arrhenius Laboratory, Stockholm University, 10 691 Stockholm, Sweden.

Materials: Bleached sulphite dissolved softwood pulp (70% Norway spruce (*Picea abeis*) and 30% Scots pine (*Pinus sylvestris*)) was received from Domsjö Fabriker AB (Sweden). D,L-Lactic acid (90%), L-Lactic acid (98%) and Hydrochloric acid (37 wt%) were purchased from VWR BDH chemicals. All chemicals were used as received without further purification. Polylactic acid 4032D (PLA): (NatureWorks, Ingeo).

Scanning Electron Microscope (SEM) images

The SEM images were recorded using FE-SEM Tescan MAIA3. Before imaging, the samples were sputtered with 5 nm Ir layer using a Quorum Q150T sputter coater.

Atomic Force Microscopy (AFM)

The AFM image was recorded on Park system NX20 instrument using NCM method (non-contact mode).

Film preparation

For film preparation, 500 ml of 0.07 wt% suspension of CNF-LA in distilled water was homogenized at 6000 rpm using an ULTRA TURRAX mixer (IKA T 25) for 30 min. The suspension was passed through a filtration system (with a fritted-glass filter support) using a membrane filter (DURAPORE 0.65 µm DVPP hydrophilic), the system was connected to vacuum, and filtration continued. The formed wet cake on the filter membrane was covered with protective paper (RKP 220 Pappersrondeller, Paper Test Equipment AB) and dried via a Rapid-Köthen sheet former (Temp.: 93 °C, applied pressure: 96 kPa, time: 10 minutes).

Water contact angle measurements (WCAs)

The static WCAs were recorded on a PGX+ Pocket Goniometer. The contact angle between the drop of water (4 μL) and the prepared film was measured on the captured image after 5 s. Measurement repeated 3 times for each sample and the average amount was reported.

Typical reaction at a 25g scale:

To a round-bottom flask (2 L), bleached sulphite pulp (25 g) and lactic acid (90%, 1 L) were subsequently added. After stirring the reaction mixture with a mechanical stirrer (1400 rpm) at 105 °C for 24 h, it was cooled to room temperature and transferred to a centrifuge vials (250 mL). Centrifugation (12000 rpm, 14 min) was followed by separation of the supernatant, which was reused for additional reaction runs (recycling), and collection of the solid material. The collected solids were washed by additional centrifugation ($3xH_20$). Next, the washed solid cellulose was dispersed into distilled water (1 L) and homogenized (IKA® T25 ULTRA TURAX, 14000 rpm) for 90 min. To determine the yield, the mixture was centrifuged (12000 rpm, 14 min) and the water was decanted and the solids were collected by lyophilization. The lyophilized solid CNF was broken down into a powder using mortar and pestle and dried under reduced pressure for 18h to give a yield of 92% (23 g).

Typical reaction at a 25g scale in the presence of HCl:

To a round-bottom flask (2 L), bleached sulphite pulp (25 g), lactic acid (90%, 1 L) and and HCl (4.3 mL, 37 wt.%, total concentration 0.05 M) were subsequently added. After stirring the reaction mixture with a mechanical stirrer (1400 rpm) at 105 °C for 24 h, it was cooled to room temperature and transferred to a centrifuge vials (250 mL). Centrifugation (12000 rpm, 14 min) was followed by separation of the supernatant, which was reused for additional reaction runs (recycling, Table S1), and collection of the solid material. The collected solids were washed by additional centrifugation ($3xH_20$). Next, the washed solid cellulose was dispersed into distilled water (1 L) and homogenized (IKA® T25 ULTRA TURAX, 14000 rpm) for 90 min. To determine the yield, the mixture was centrifuged (12000 rpm, 14 min) and the water was decanted and the solids were collected by lyophilization. The lyophilized solid CNF was broken down into a powder using mortar and pestle and dried under reduced pressure for 18h to give a yield of 72% (18 g).

Table S1. Recycling and yields at 25g scale

Reactions	Yields%
1st	72
2nd	76
3rd	82
4th	85
5th	84
6th	86
7th	87

Hydrolysis of LA-CNF to obtain CNF:

LA-CNFs (0.5 g) fabricated using no HCl was dispersed in 40 mL of EtOH (*aq*) 70% v/v, then 30 mL of NaOH (0.5 M) was added dropwise and the mixture was stirred at 60 °C for 24 h. After cooling down to room temperature, the mixture was centrifugated (12000 rpm, 14 min), and the solid materials were collected. The collected solids were re-dispersed in water and washed by centrifugation (3x50 mL H_20).

Micro-compounding and tensile tests:

The fabricated CNFs materials (LA-CNF and CNF) were mixed with PLA (melt-blending) using a twin-screw micro-compounder (Xplore, 15 mL) at 190 °C, first at 10 rpm for around 11 min and then at 100 rpm and under nitrogen for 3 min.

Using Xplore IM 5.5 micro injector, dog bone shape specimens for tensile tests were made via injection molding technique, (injection temperature 195 °C, applies pressure 7 bar, and temperature of mold 50 °C). The specimens were kept in conditioning room for 24 h and tensile tests were carried out using Zwick Z2.5 (ZwickRoell Ltd., UK) with the rate of 2.5 mm/min.

Differential Scanning Calorimetry (DSC) analysis:

DSC was performed on melt processed materials, with a Mettler Toledo DSC2 machine using Aluminium 40 µl pans. The analysis followed a heating/cooling/heating temperature profile from -80 °C to 250 °C, at a heating rate of 10 °C/min, under N_2 constant flow of 50 ml/min.

Addition of CNF and LA-CNF to PLA:

The addition of CNF increases the tensile strength of PLA by 9 % while LA-CNF shows a slight improvement in the Young's modulus (Table S2). The elongation at break of PLA is not affected by the nanofibrils, suggesting a good dispersion. Overall, the mechanical properties are not greatly influenced possibly due to the low amounts of nanofibrils in the composites and to the already stiff and brittle nature of PLA. The DSC analysis shows that the glass transition and temperatures of PLA are also not affected (Figure S1). PLA is characterized by two melting peaks at 162 and 168 °C related to α′ and α crystalline forms, respectively. The inclusion of 1 wt.% CNF of LA-CNF appears to increase the β form while at 2 wt.% of nanofibrils the α form is predominant. The largest impact is observed for 2 wt.% LA-CNF in which α form of crystals is prevalent and consistently the cold crystallization temperature of PLA is lowered. Despite the nucleating effect of the nanofibrils, which lower the crystallization temperature, PLA remains amorphous.

Table S2. Tensile strength and young's modulus of PLA with and without adding CNF components.

Composition [a]	Tensile strength (MPa)	Young's modulus (MPa)	Elongation at break (%)
1) PLA (neat)	66	1020	9
2) PLA and CNF (2 %wt.)	72	950	10
3) PLA with CNF (1 %wt.)	72	1000	10
4) PLA+LA-CNF (2 %wt.)	69	1070	10
5) PLA+LA-CNF (1 %wt.)	69	1040	11

a) Weight % of CNF in parenthesis

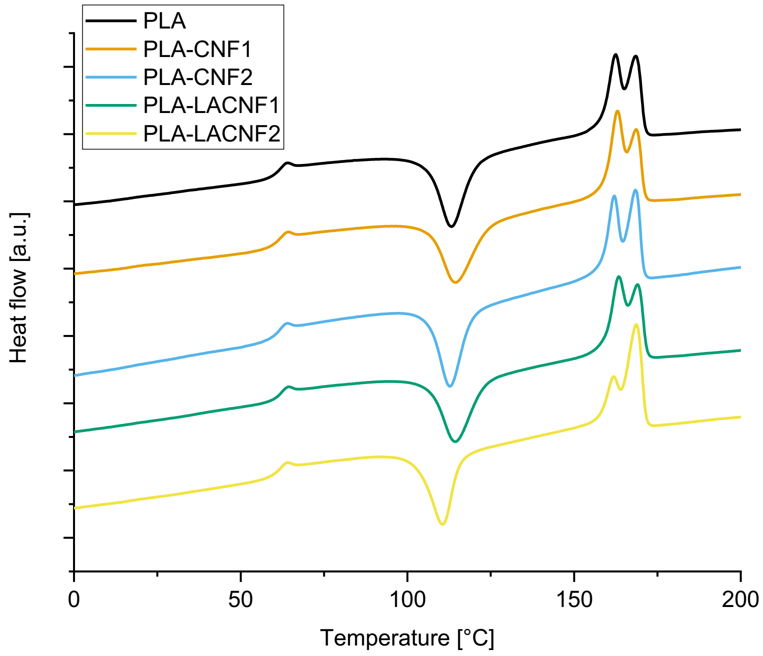

Figure S1. Second heating scan of DSC.

Table S3. Thermal properties of the materials measured by DSC. ΔH_{cc} is the enthalpy of cold crystallization.

Composition	ΔH_{cc} (J/g)	ΔH_m (J/g)	PLA_f	χ_c (%)	T_{m1} (°C)	T_{m2} (°C)	T_{cc} (°C)	T_g (°C)
PLA	-40.11	40.28	1	0	162	168	113	62
PLA-CNF1	-36.04	38.82	0.99	3	163	169	115	62
PLA-CNF2	-37.82	39.43	0.98	2	162	168	113	62
PLA-LACNF1	-37.23	37.63	0.99	0	163	169	114	62
PLA-LACNF2	-36.69	37.55	0.98	1	162	169	110	62

ΔH_m is the melting enthalpy; PLAf is the weight fraction of PLA in the materials; χ_c is the crystallinity measured considering a ΔH_{m0} of 93 J/g [1]for neat PLA[1]; T_{m1} and T_{m2} are the two melting peaks; T_{cc} is the cold crystallization temperature and T_g is the glass transition temperature.

References:

1) Zaldua, N. et al. *CrystEngComm*. **18**, 9334-9344 (2016).

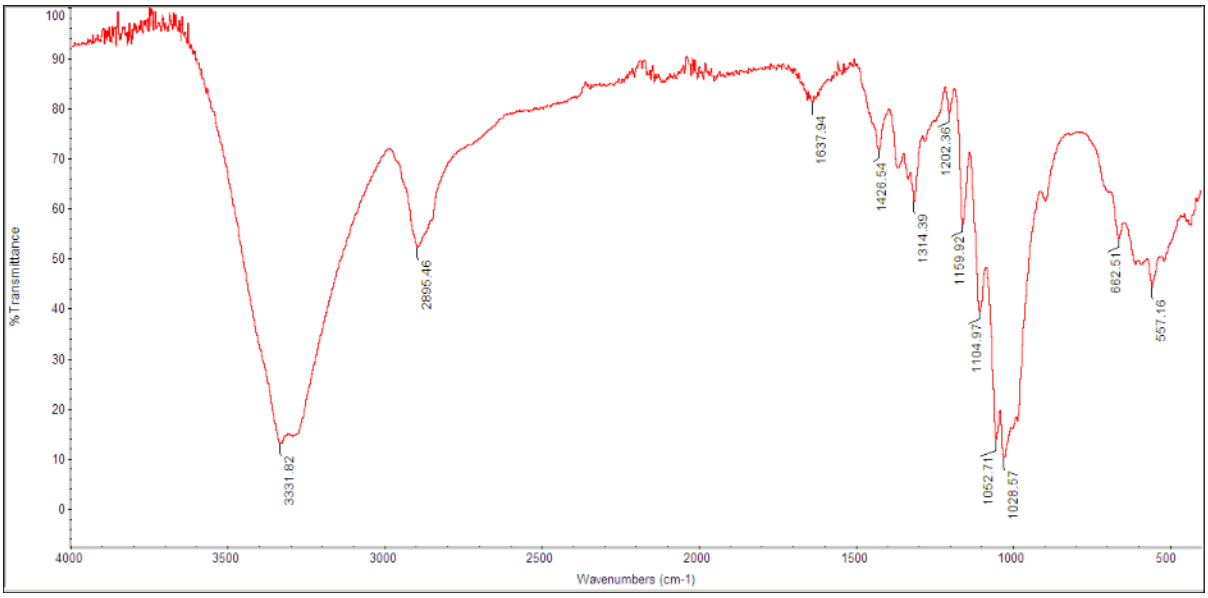

Fig. S2. FTIR spectrum of cellulose starting material (sulphite pulp)

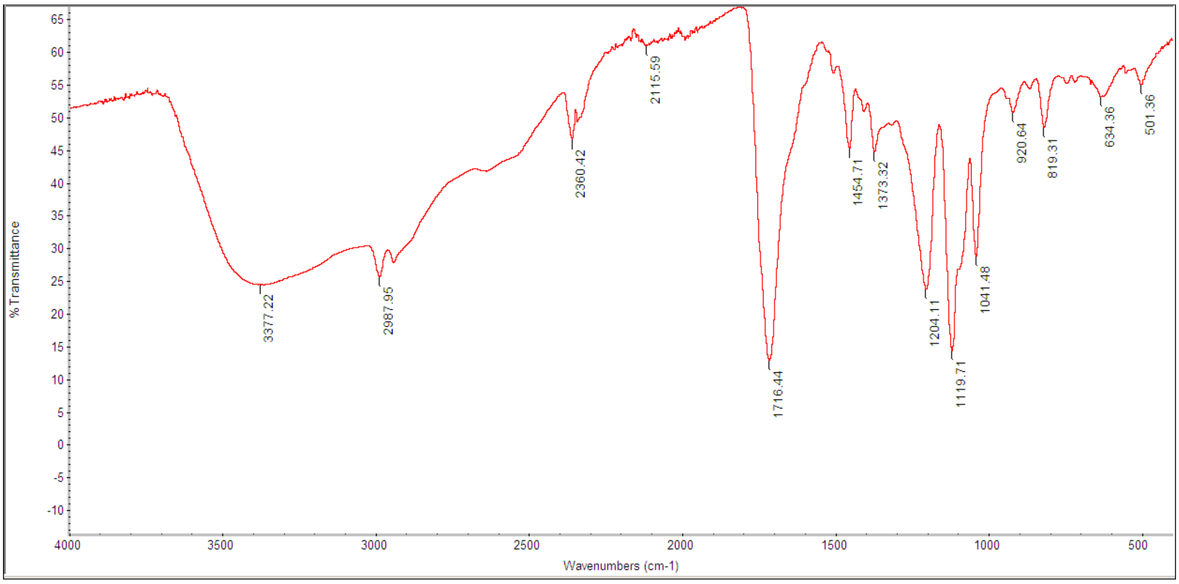

Fig. S3. FTIR spectrum of D,L-Lactic acid

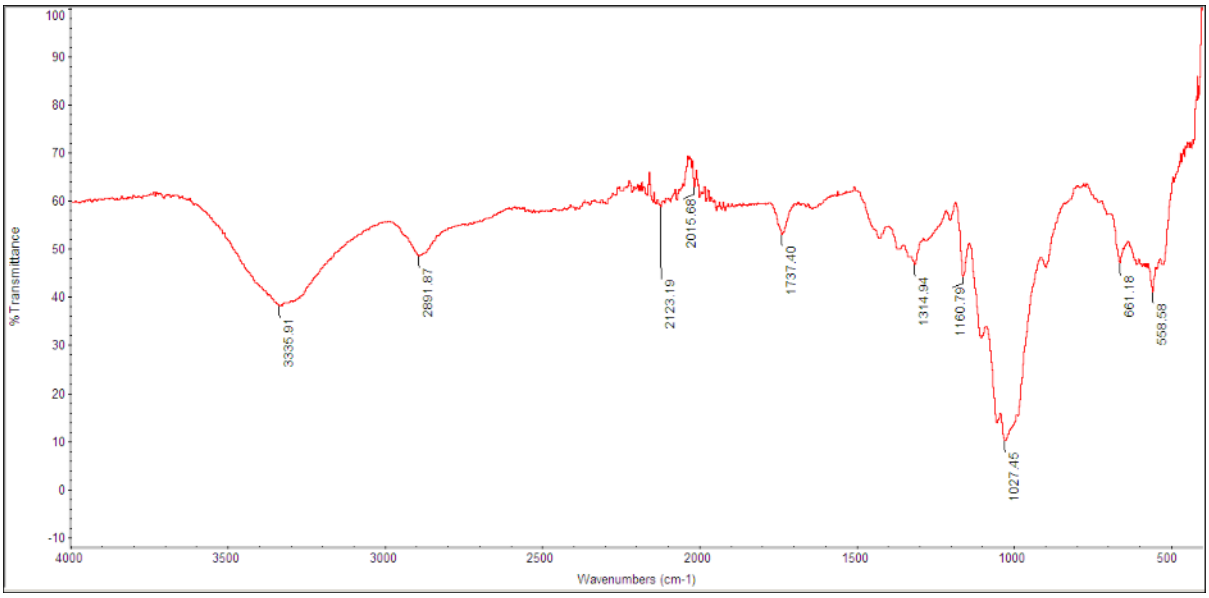

Fig. S4. FTIR spectrum of LA-functionalized CNF (using HCl 0,05M)

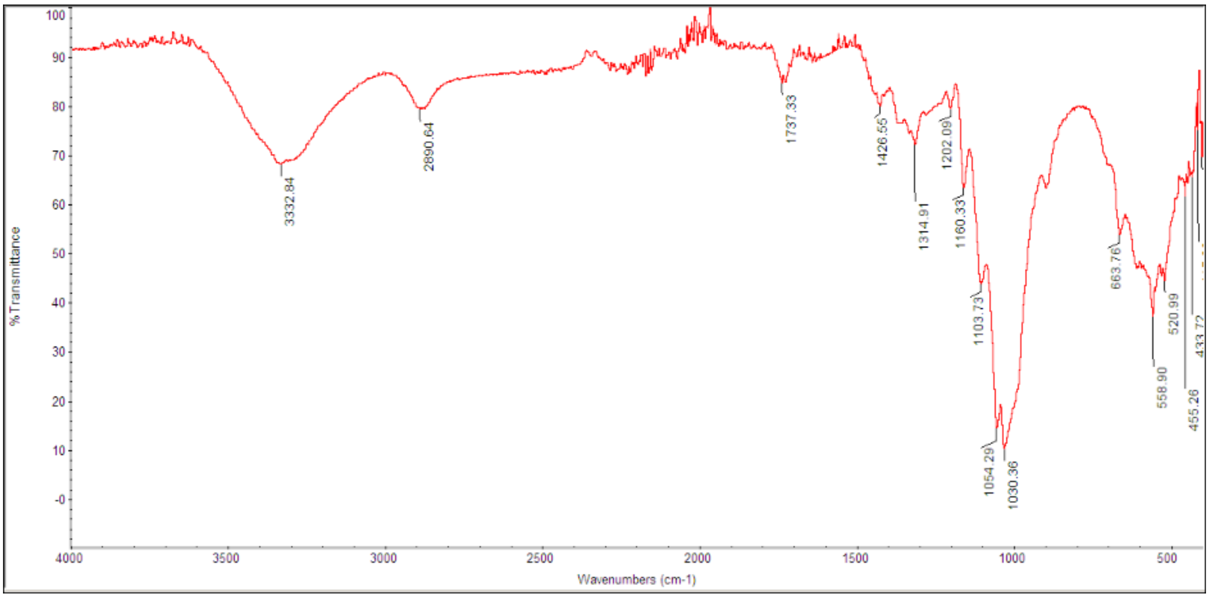

Fig. S5. FTIR spectrum of LA-functionalized CNFs (using HCl 0,1M)

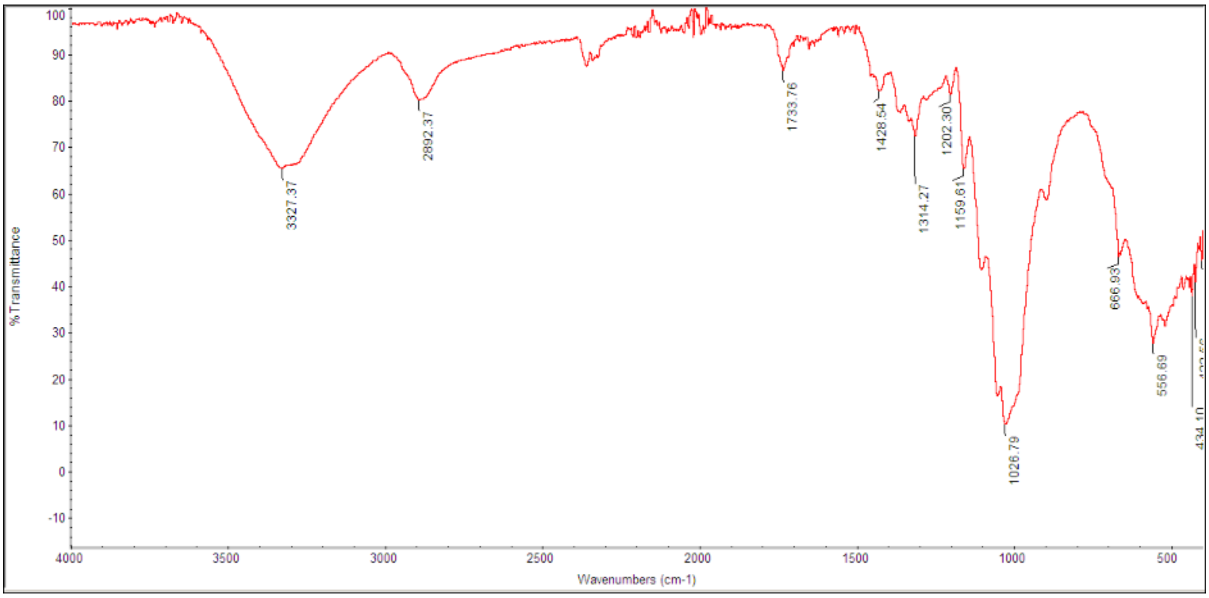

Fig. S6. FTIR spectrum of LA-functionalized CNFs (using No HCl)

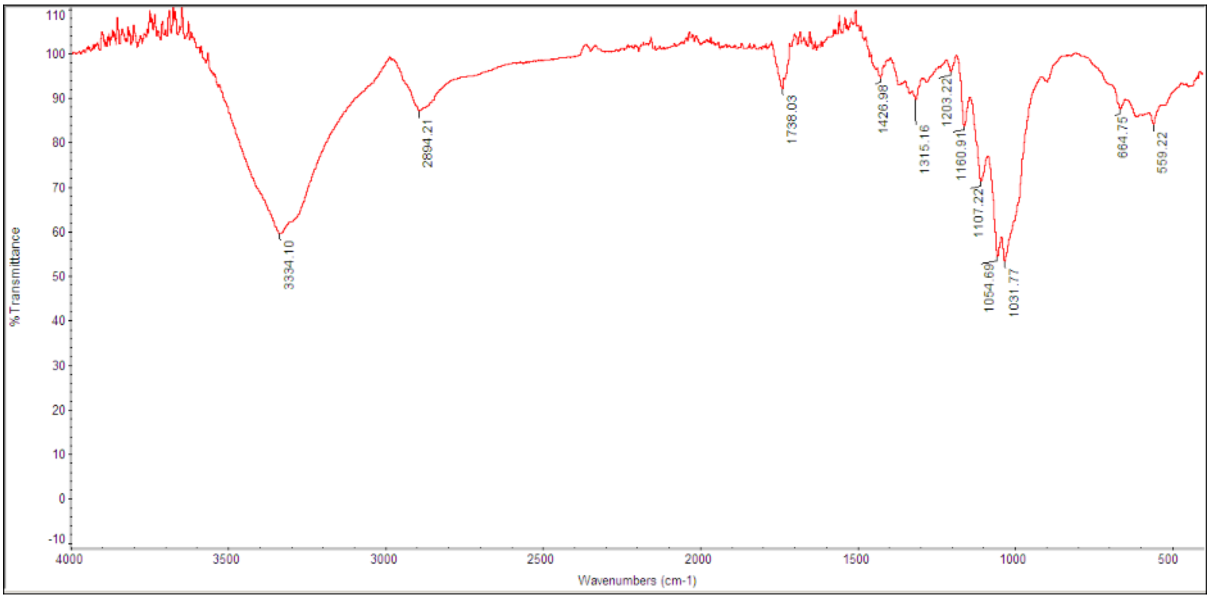

Fig. S7. FTIR spectrum of L-LA functionalized CNFs (using L-Lactic acid)

FTIR spectra of LA functionalized CNFs using recycled lactic acid media (without HCl, Fig. S8-S14)

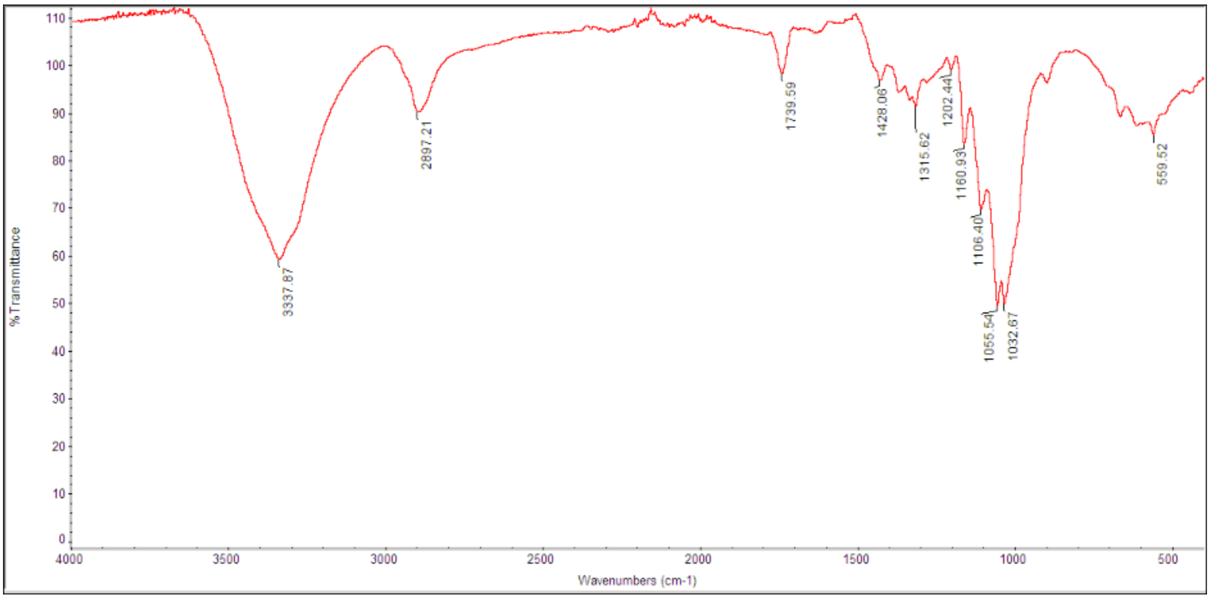

Fig. S8. FTIR spectrum of LA functionalized CNFs using recycled LA (2nd reaction).

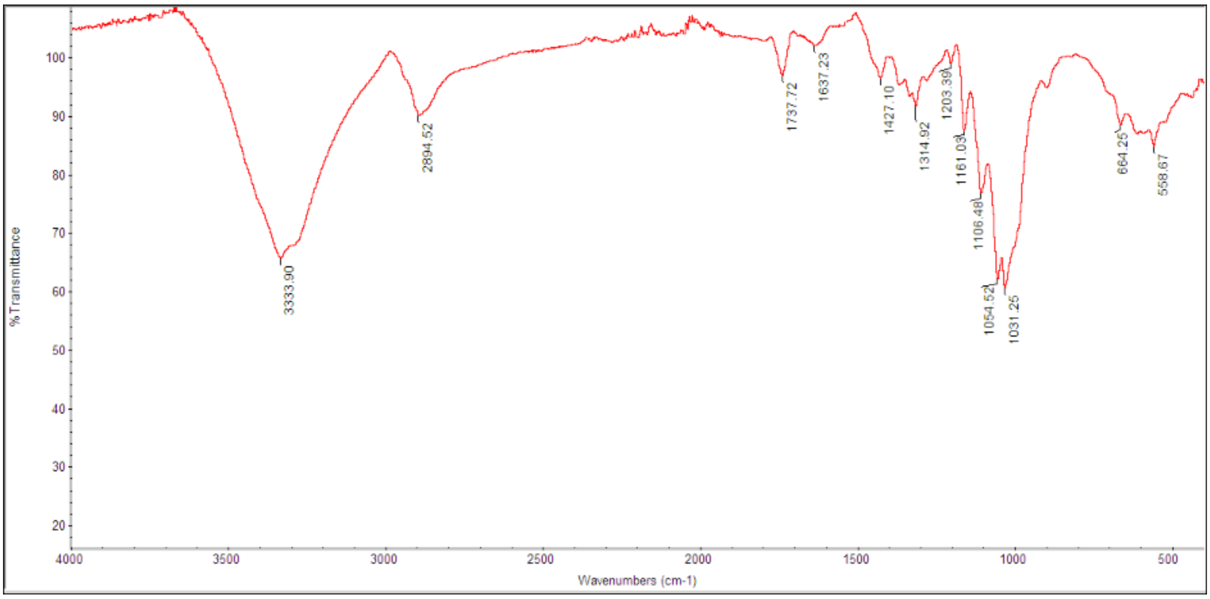

Fig. S9. FTIR spectrum of LA functionalized CNFs using recycled LA (3rd reaction).

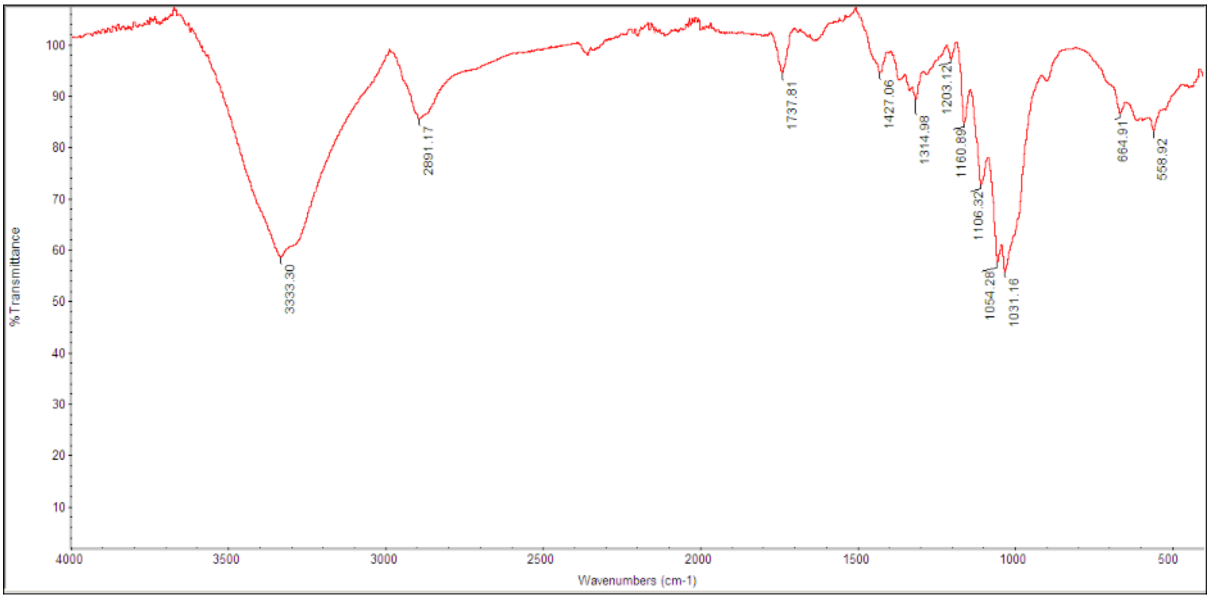

Fig. S10. FTIR spectrum of LA functionalized CNFs using recycled LA (4th reaction).

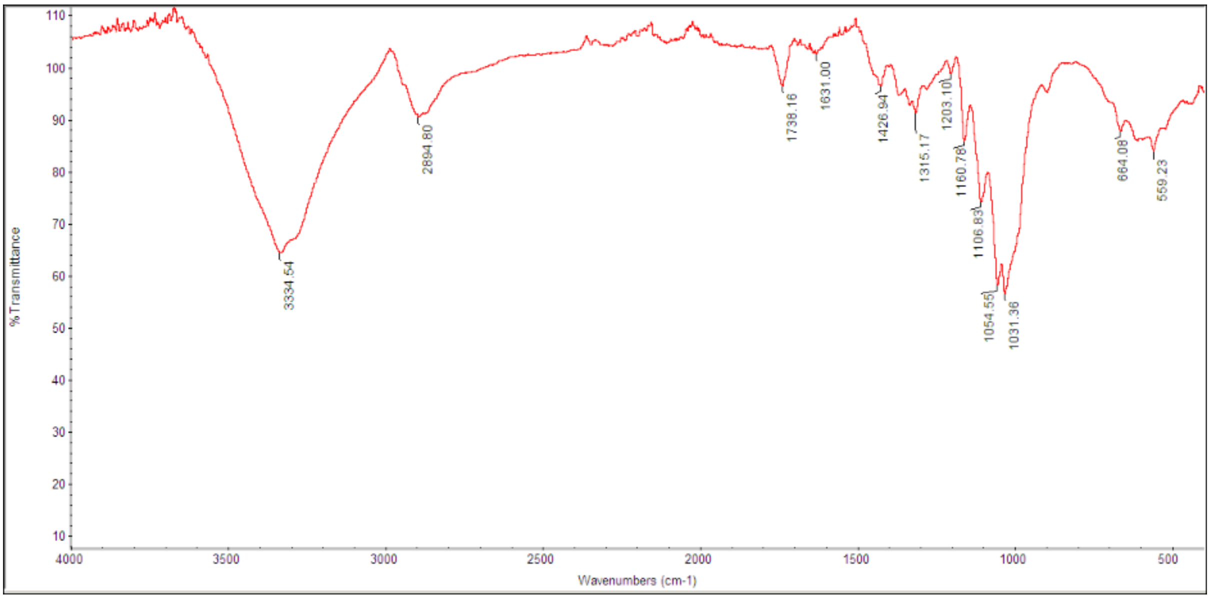

Fig. S11. FTIR spectrum of LA functionalized CNFs using recycled LA (5th reaction).

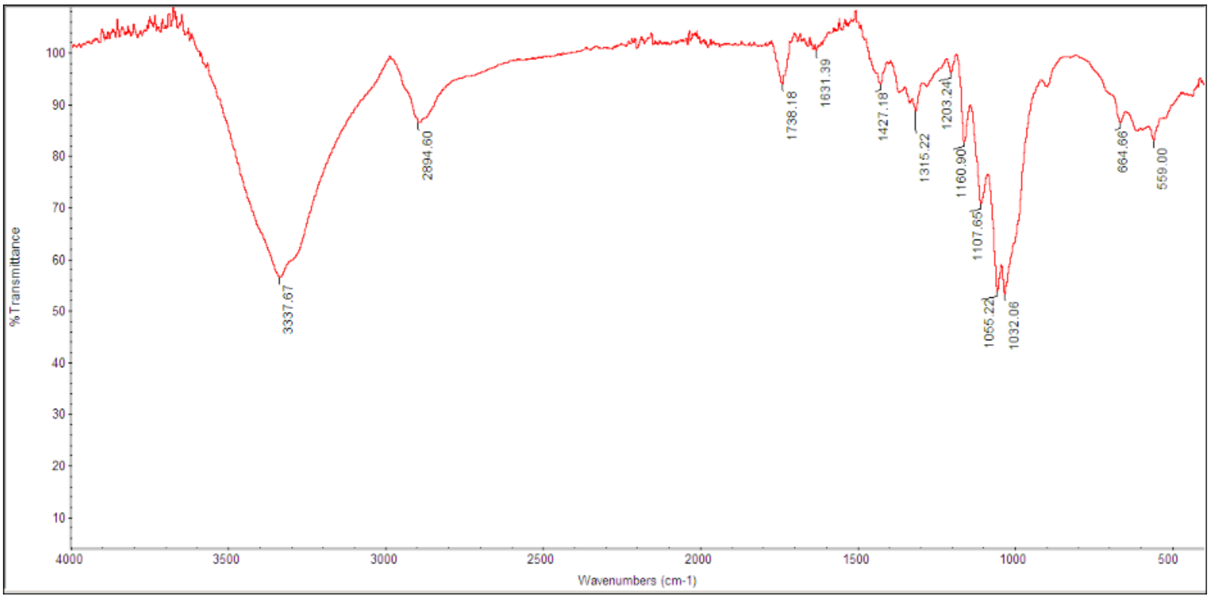

Fig. S12. FTIR spectrum of LA functionalized CNFs using recycled LA (6nd reaction).

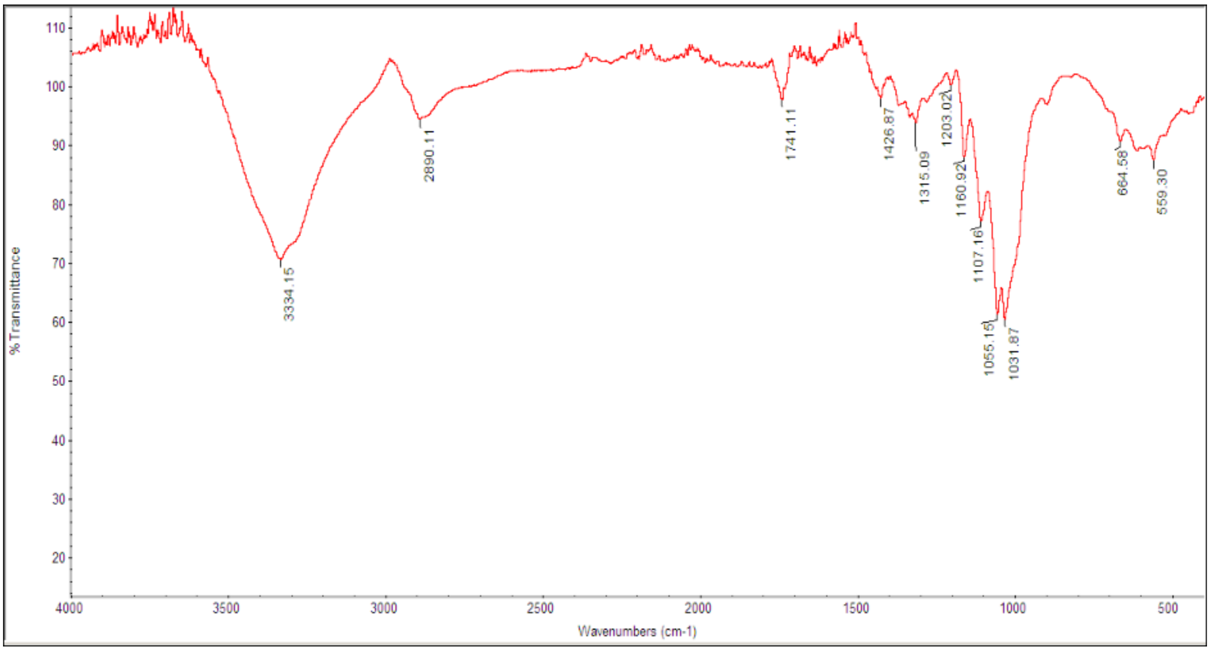

Fig. S13. FTIR spectrum of LA functionalized CNFs using recycled LA (7th reaction).

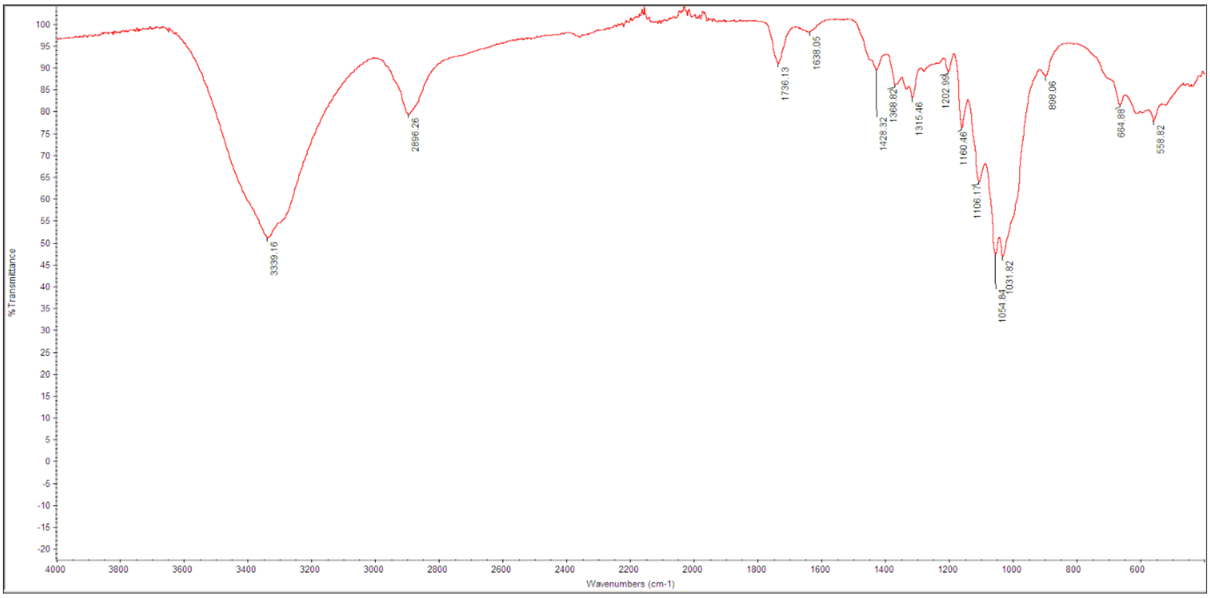

Fig. S14. FTIR spectrum of LA functionalized CNFs using recycled LA (8th reaction).

FTIR spectra of LA functionalized CNFs using recycled lactic acid media (lactic acid + HCl 0.05M; Fig. S14-S18)

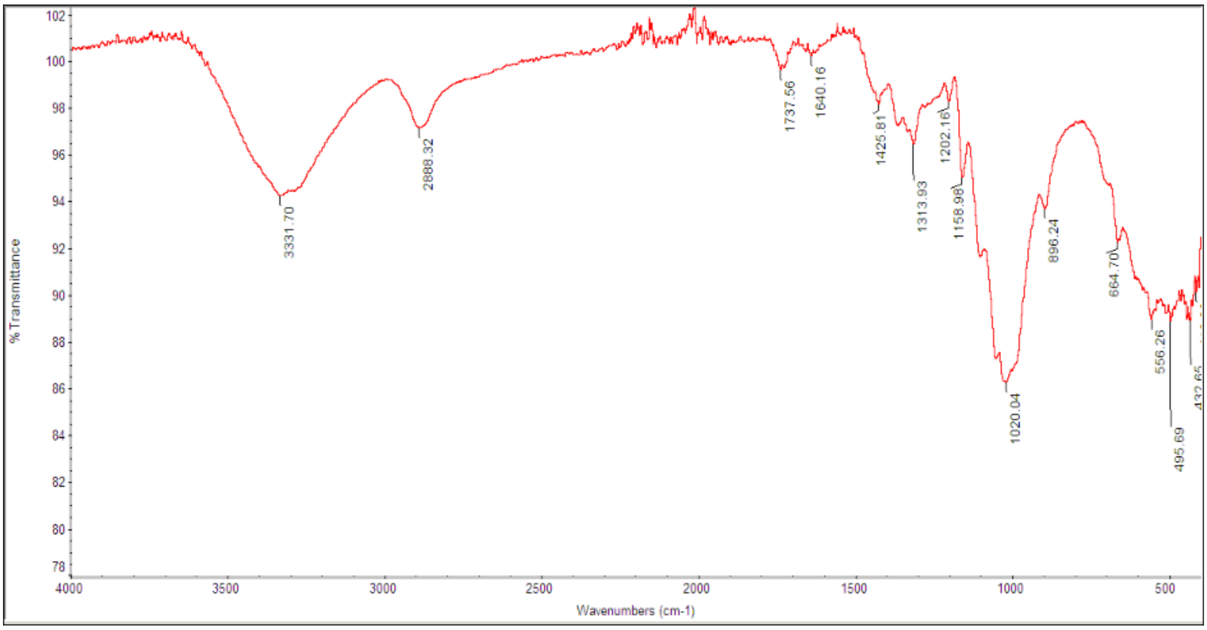

Fig. S14. FTIR spectrum of LA functionalized CNFs using recycled LA (2nd reaction).

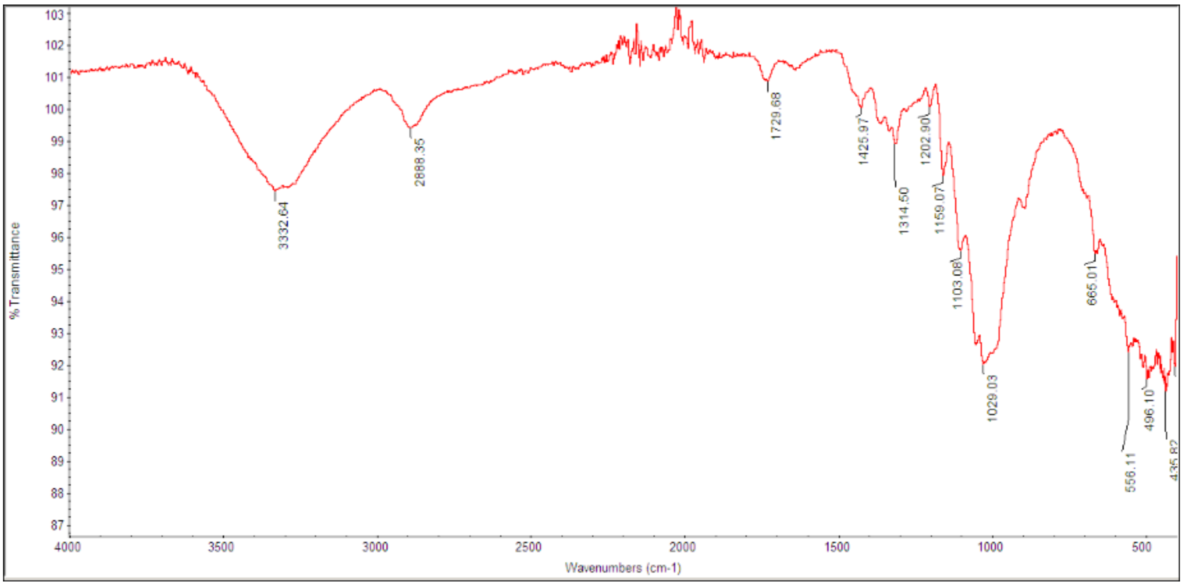

Fig. S15. FTIR spectrum of LA functionalized CNFs using recycled LA (3rd reaction).

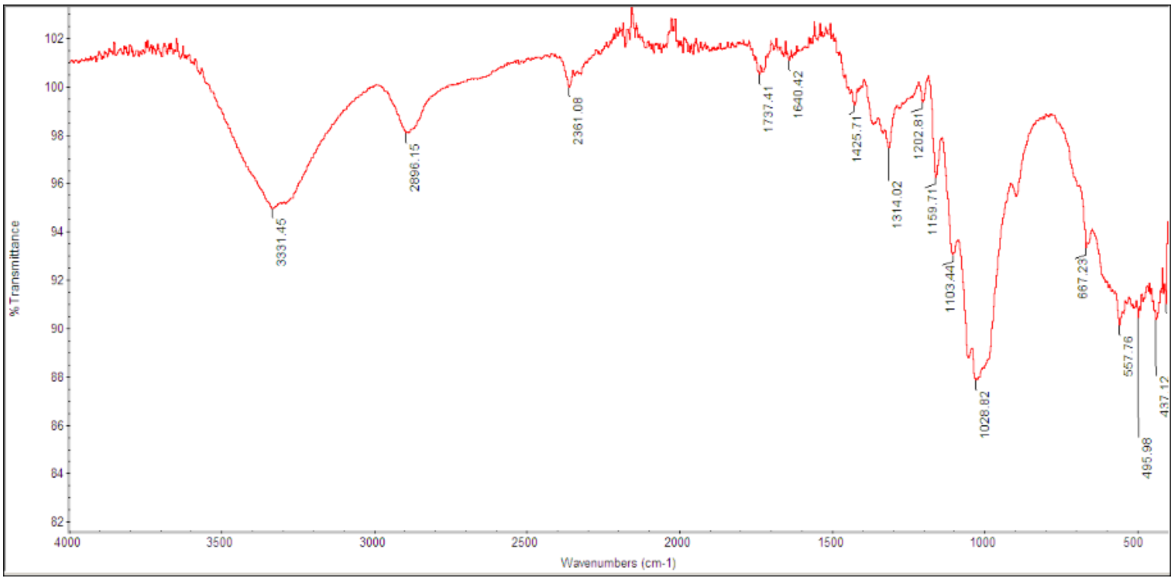

Fig. S16. FTIR spectrum of LA functionalized CNFs using recycled LA (4th reaction).

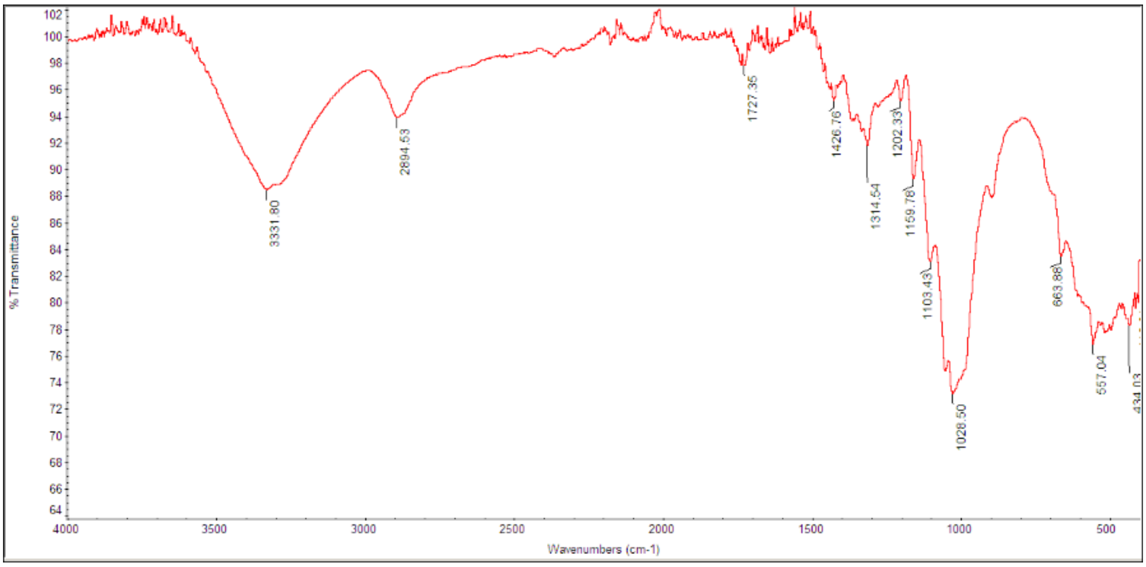

Fig. S17. FTIR spectrum of LA functionalized CNFs using recycled LA (5th reaction).

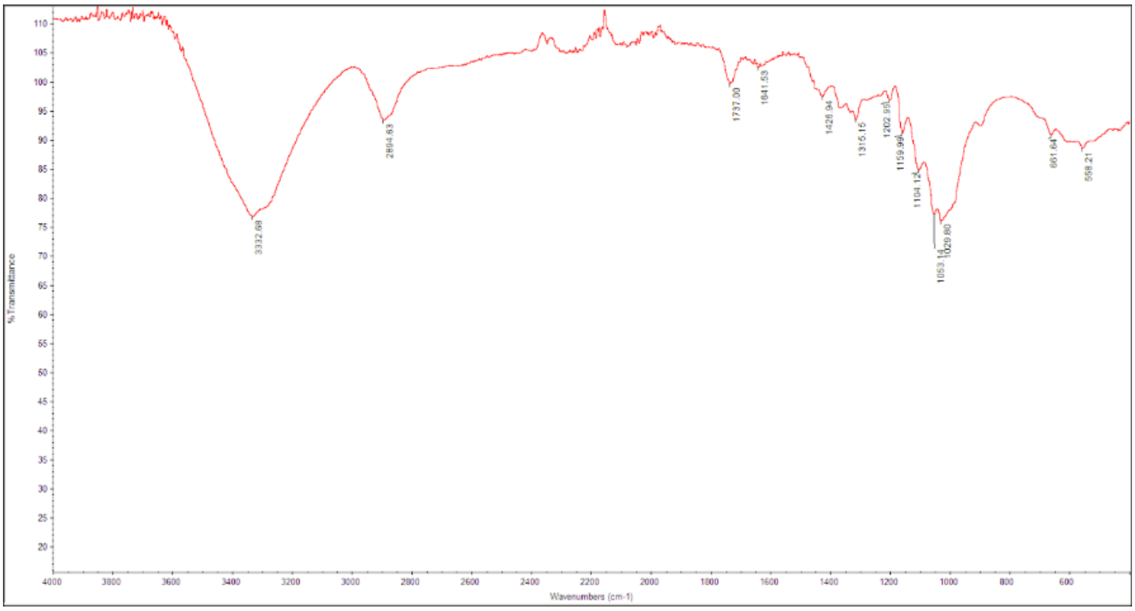

Fig. S18. FTIR spectrum of LA functionalized CNFs using recycled LA (6th reaction).

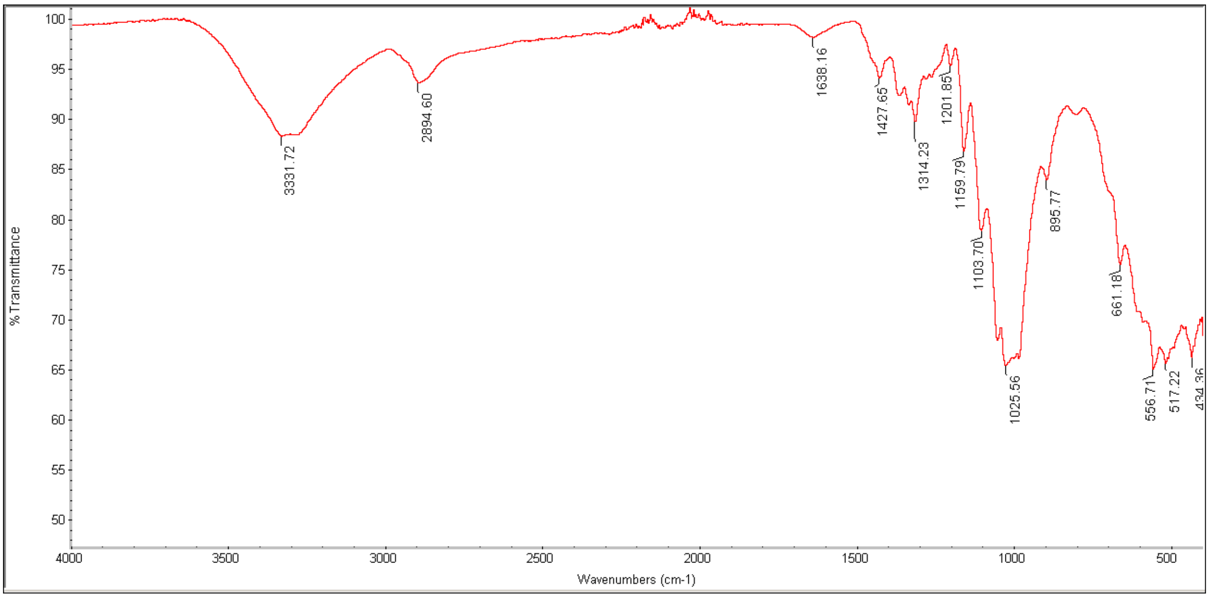

Fig. S19. FTIR spectrum of film made of alkali hydrolyzed LA functionalized CNFs.

The HNMR of Lactic acid before reaction and lactic acid solution after 5 reactions looks the same.

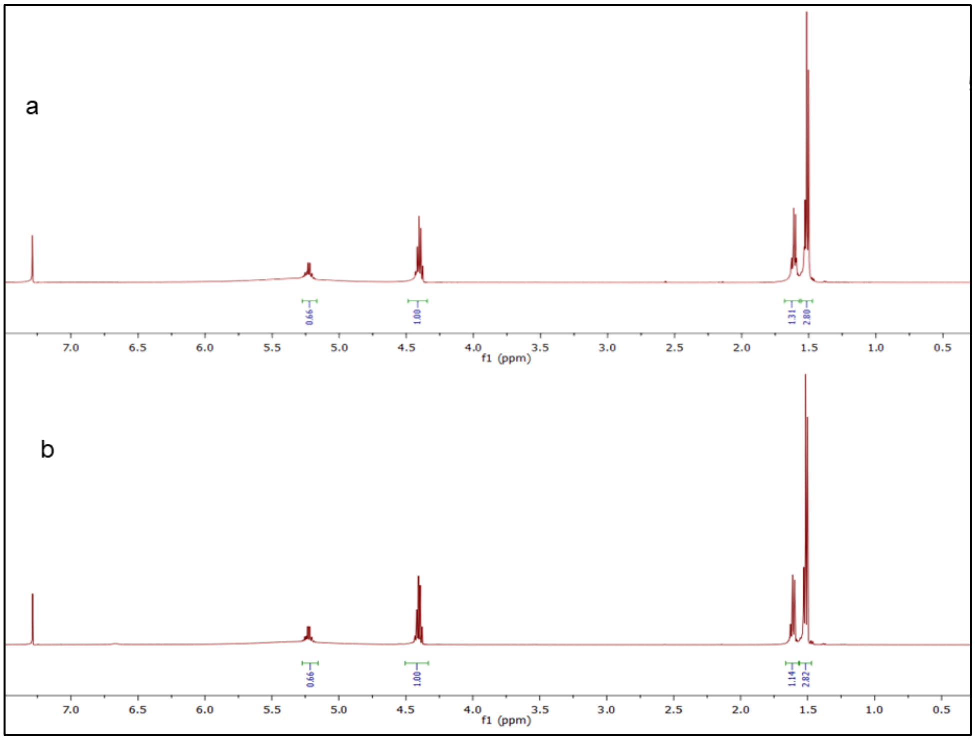

Fig. S20. The HNMR of lactic acid media used for 6 times (spectrum a) and commercial D,L-Lactic acid (spectrum b) in $CDCl_3$.

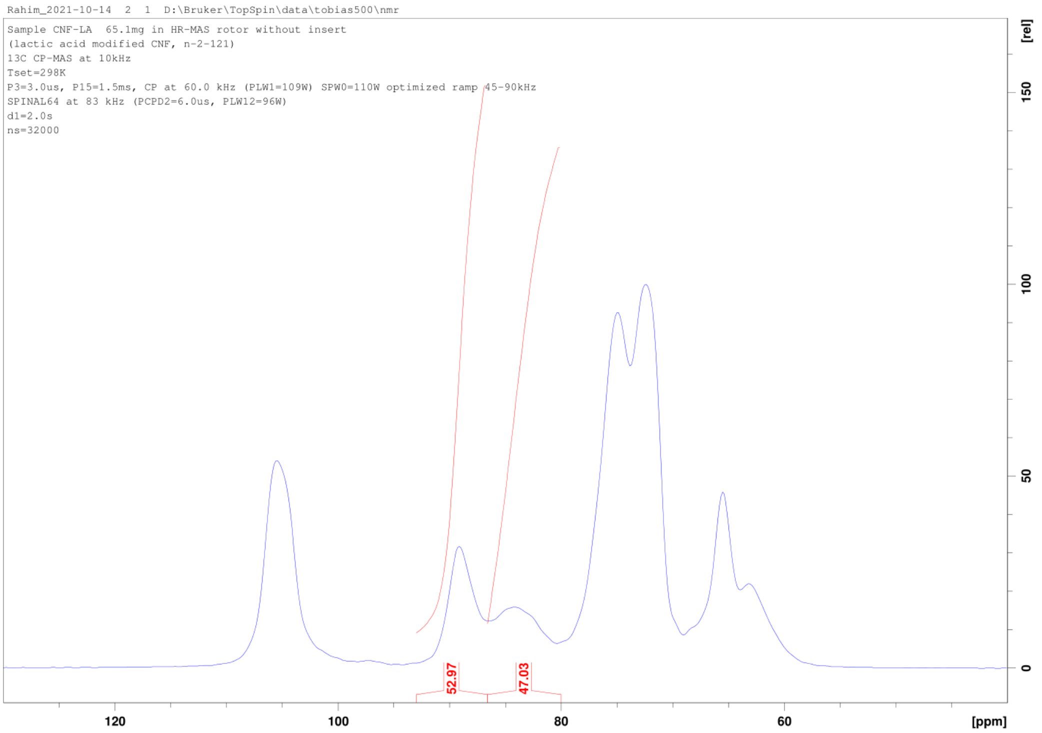

Fig. S21. Solid state CP/MAS ^{13}C NMR spectrum of LA functionalized CNFs (using 0,1 M HCl).

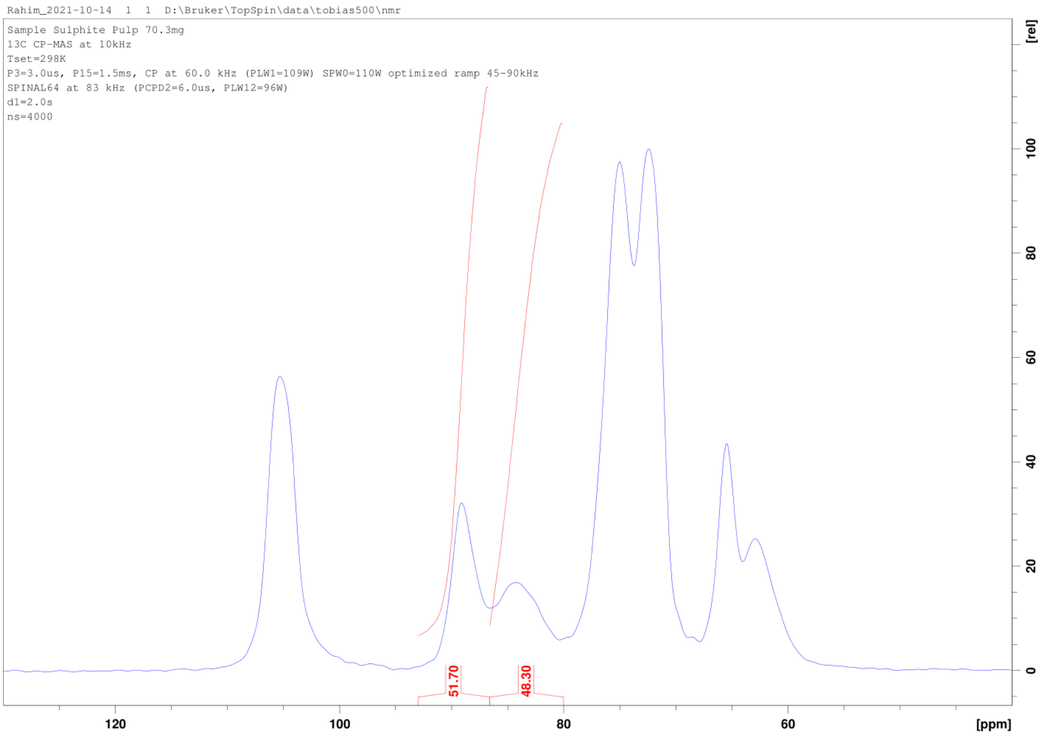

Fig. S22. Solid state CP/MAS ^{13}C NMR spectrum of sulphite pulp as starting material.

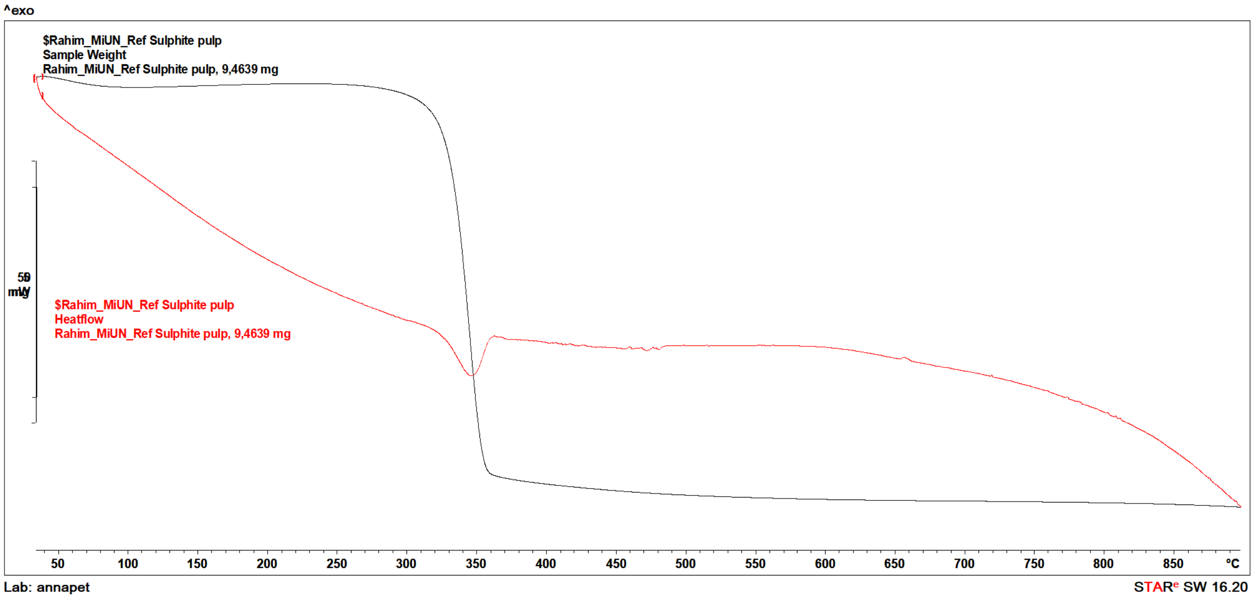

Fig. S23. TGA curve of sulphite pulp

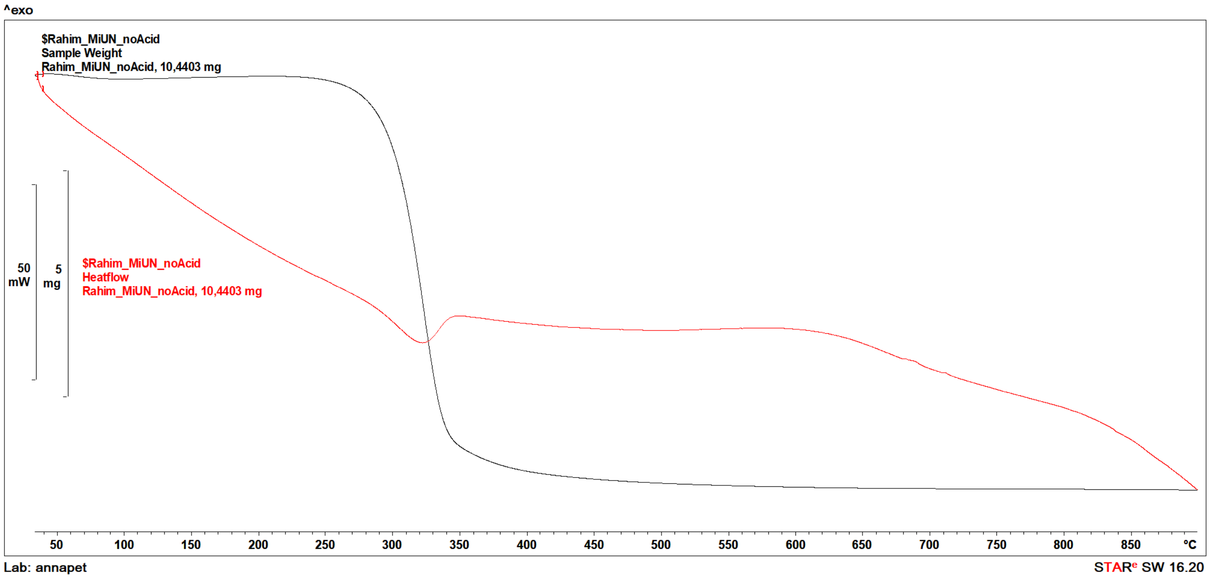

Fig. S24. TGA curve of LA functionalized CNF (using LA without adding HCl)

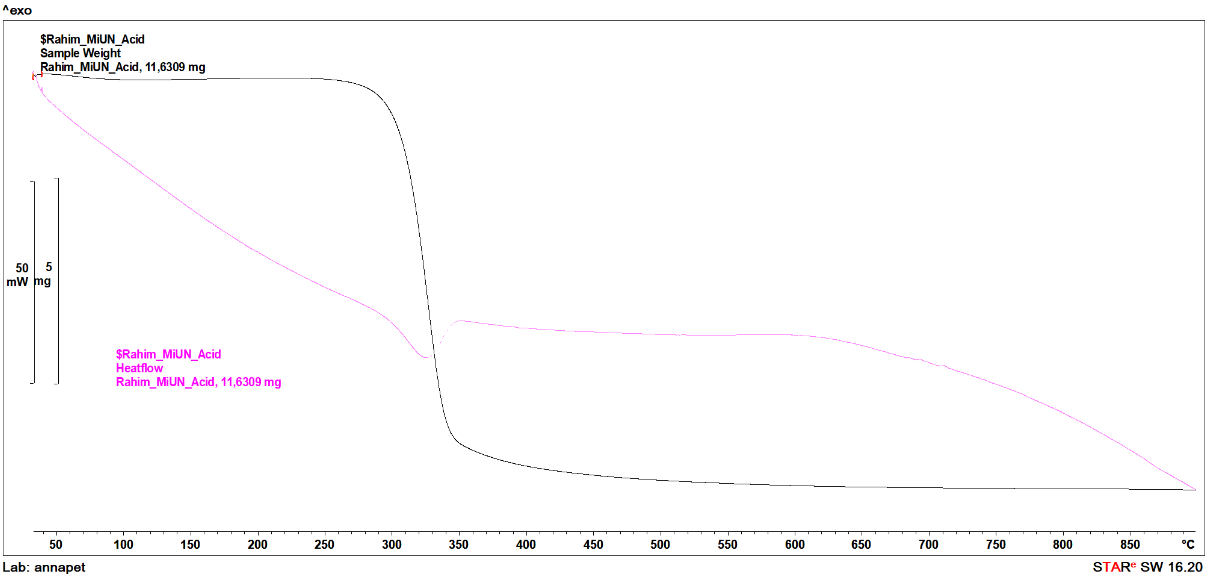

Fig. S25. TGA curve of LA functionalized CNF (using LA+HCl 0.1 M)

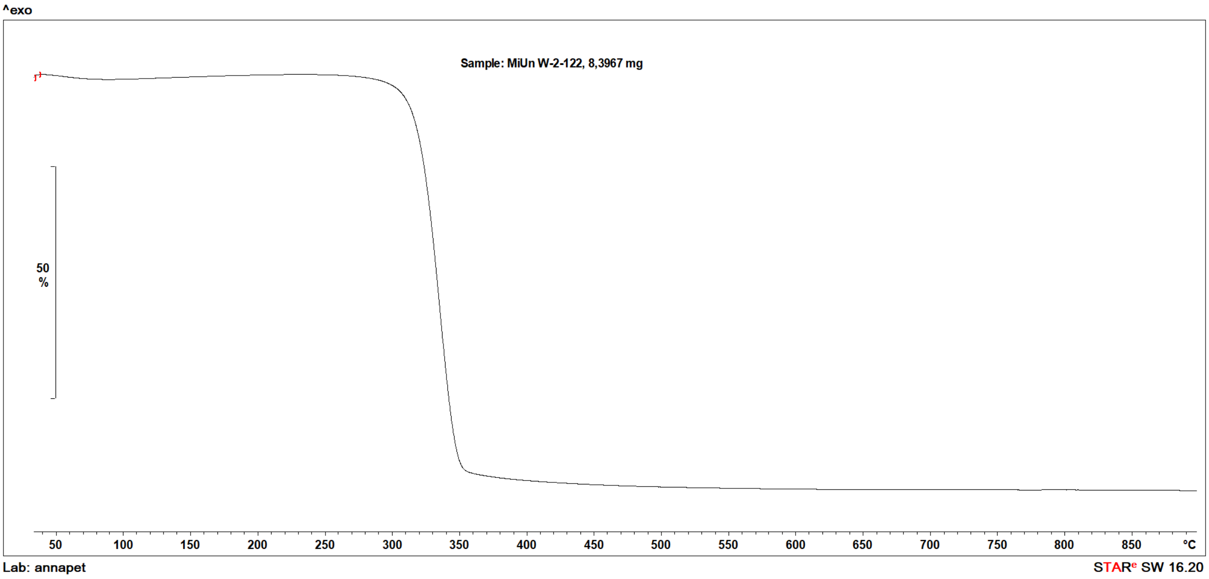

Fig. S26. TGA curve of LA functionalized using LA without adding HCl (before homogenization)

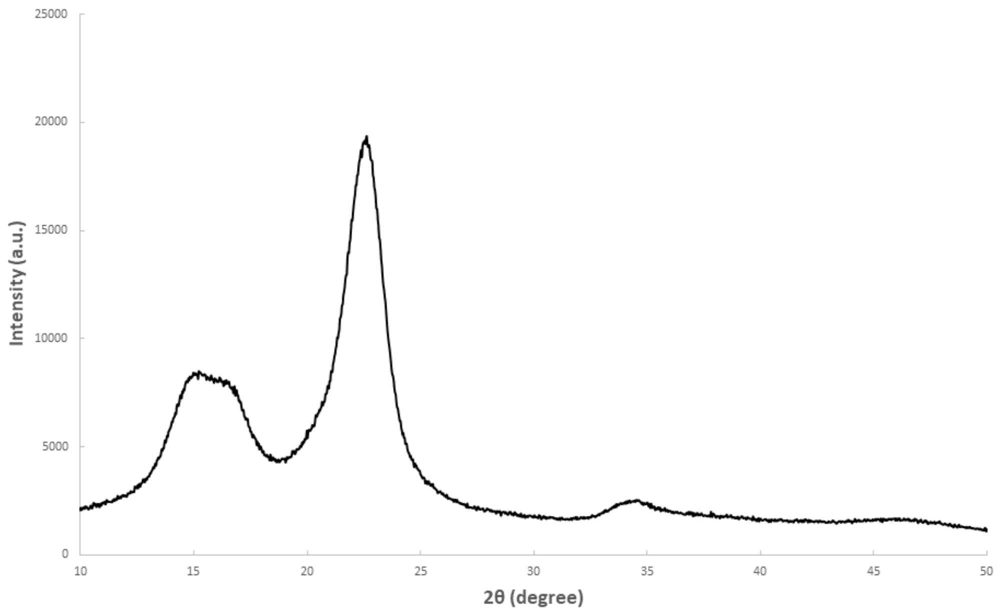

Fig. S27. XRD spectrum of cellulose starting material (sulphite pulp)

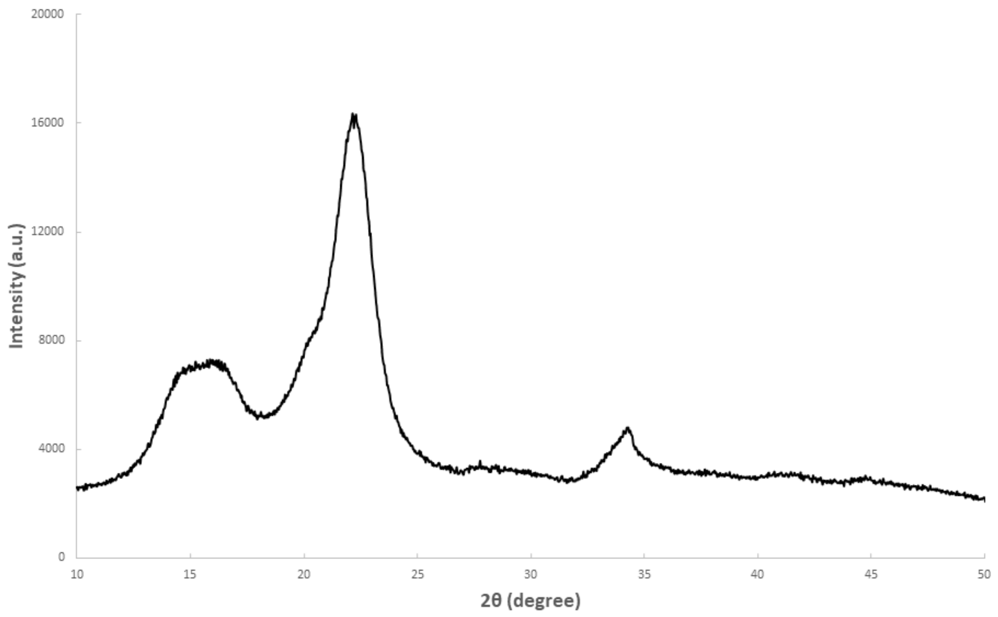

Fig. S28. XRD spectrum of LA functionalized CNF (using LA+HCl 0.1 M)

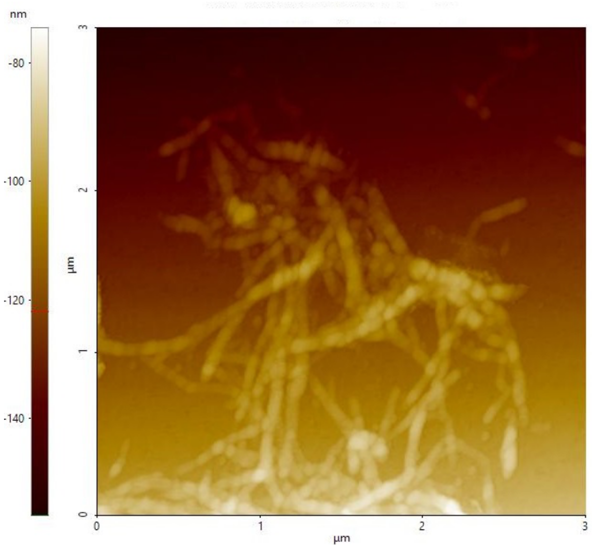

Fig. S29. AFM image of LA functionalized CNFs fabricated with lactic acid (without adding HCl).

Molecular Weight Averages

Peak	Mp (g/mol)	Mn (g/mol)	Mw (g/mol)	Mz (g/mol)	Mz+1 (g/mol)	Mv (g/mol)	PD
Peak 1	64783	21142	112639	325120	603558	289584	5.328

Distribution Plot

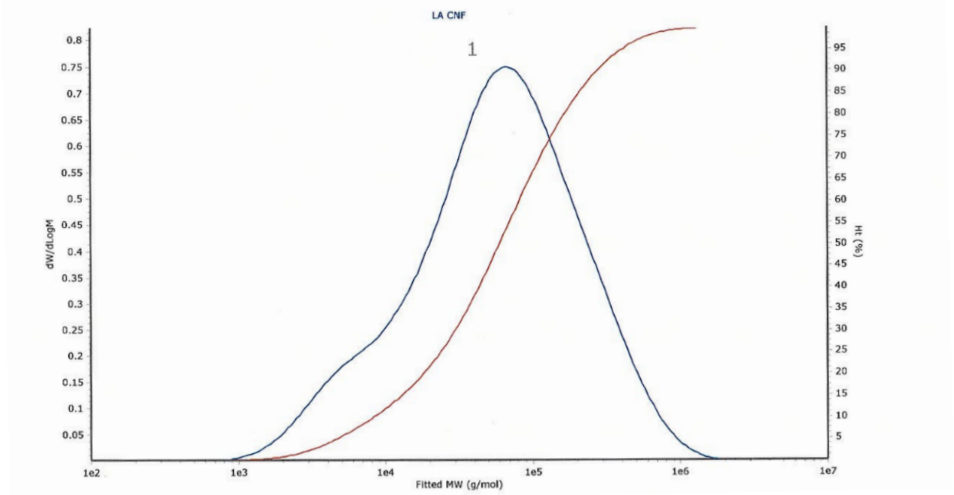

Fig. S30. MWD curve (from SEC analysis) of LA functionalized CNF (using no HCl).

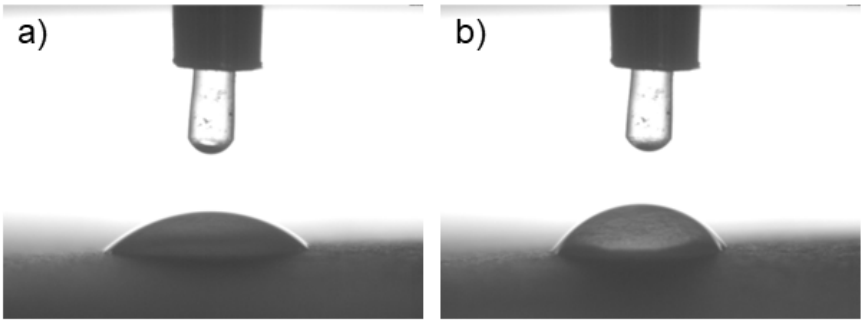

Fig. S31. Water contact angle pictures of the prepared films; a) film made of hydrolyzed LA functionalized CNF (42±3°); b) film made of LA functionalized CNF (58±1°).

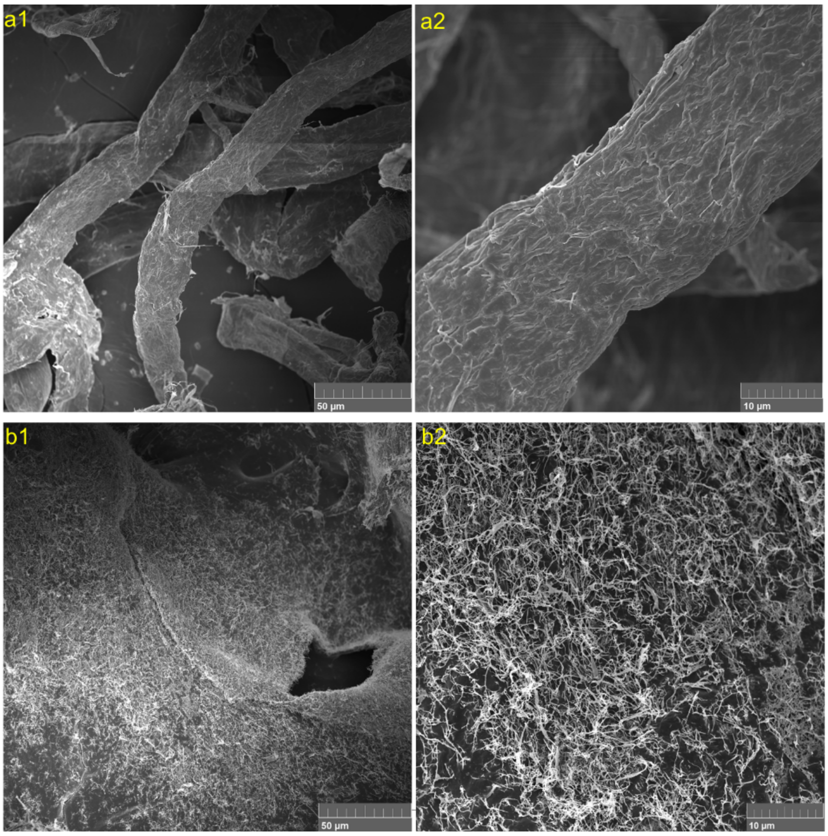

Fig. S32. SEM images: (a1) Sulphite pulp at 50 μm. (a2) Sulphite pulp at 10 μm. (b1) LA-functionalized CNFs fabricated with lactic acid (without adding HCl). (b2) LA-functionalized CNFs fabricated with lactic acid (without adding HCl).
